# Sequential Paleotetraploidization shaped the carrot genome

**DOI:** 10.1186/s12870-020-2235-7

**Published:** 2020-01-31

**Authors:** Jinpeng Wang, Jigao Yu, Yuxian Li, Chendan Wei, He Guo, Ying Liu, Jin Zhang, Xiuqing Li, Xiyin Wang

**Affiliations:** 1 Center for Genomics and Computational Biology, School of Life Sciences, North China University of Science and Technology, Tangshan, 063200 Hebei China; 2 State Key Laboratory of Systematic and Evolutionary Botany, Institute of Botany, Chinese Academy of Sciences, Beijing, 100093 China; 3 University of Chinese Academy of Sciences, Beijing, 100049 China; 4 Fredericton Research and Development Centre, Agriculture and Agri-Food Canada, Fredericton, Frederiction, New Brunswick E3B 4Z7 Canada; 5 School of Genomics and Bio-Big-Data, Chengdu University of Traditional Chinese Medicine, Chengdu, 610075 China; 6 College of Mathematics and Science, Handan University, Handan, 056005 Hebei China

**Keywords:** Carrot, Coffee, Lettuce, Carotenoids, Gene collinearity, Genomic fractionation, Polyploidization

## Abstract

**Background:**

Carrot (*Daucus carota subsp. carota* L.) is an important root crop with an available high-quality genome. The carrot genome is thought to have undergone recursive paleo-polyploidization, but the extent, occurrences, and nature of these events are not clearly defined.

**Results:**

Using a previously published comparative genomics pipeline, we reanalysed the carrot genome and characterized genomic fractionation, as well as gene loss and retention, after each of the two tetraploidization events and inferred a dominant and sensitive subgenome for each event. In particular, we found strong evidence of two sequential tetraploidization events, with one (Dc-α) approximately 46–52 million years ago (Mya) and the other (Dc-β) approximately 77–87 Mya, both likely allotetraploidization in nature. The Dc-β event was likely common to all Apiales plants, occurring around the divergence of Apiales-Bruniales and after the divergence of Apiales-Asterales, likely playing an important role in the derivation and divergence of Apiales species. Furthermore, we found that rounds of polyploidy events contributed to the expansion of gene families responsible for plastidial methylerythritol phosphate (MEP), the precursor of carotenoid accumulation, and shaped underlying regulatory pathways. The alignment of orthologous and paralogous genes related to different events of polyploidization and speciation constitutes a comparative genomics platform for studying Apiales, Asterales, and many other related species.

**Conclusions:**

Hierarchical inference of homology revealed two tetraploidization events that shaped the carrot genome, which likely contributed to the successful establishment of Apiales plants and the expansion of MEP, upstream of the carotenoid accumulation pathway.

## Background

*Daucus carota subsp. carota* L. (carrot) is one of the most important vegetable crops because it is a main source of vitamin A and carotenoids [1, 2]. *Daucus c. carota* belongs to the Apiaceae family within the order Apiales, within the Campanulids clade, which also includes the order Asterales (with key species such as *Lactuca sativa* L. or *Helianthus annuus* L.) [3]. The Lamiids, a close sister clade of Campanulids, encompasses many species of agricultural importance that are distributed in several orders, such as Gentianales (e.g., *Coffea canephora* Pierre ex A. Froehner, *Swertia bimaculate* (Siebold & Zucc.) Hook. F. & Thomson ex C.B. Clarke) or Solanales (e.g., *Solanum muricatum* Aiton, *Solanum tuberosum* L.) [4]. Both Campanulids and Lamiids clades belong to the Asterids clade, a sister group of Rosids (e.g., *Vitis vinifera* L.) within the Eudicots clade [5].

Ancient polyploidization events have played important roles in the evolution of land plants, contributing to their origin and diversification [6–10]. Carrot was the first Apiaceae species to be completely sequenced. By genome comparison, it was stated that the carrot genome may have been affected by two polyploidization events, previously referred to as Dc-α and Dc-β, likely resulting in a whole-genome triplication (× 3) and a whole-genome duplication event (× 2) [11], respectively. However, a detailed interpretation of the order, occurrence, and the resulting separation of duplicated genes produced by these events has remained elusive. This is largely due to the complexity of the carrot genome, which has undergone recursive rounds of polyploidization.

In addition to the abovementioned events, carrot and other eudicots (e.g., coffee and grape) shared a more ancient core-eudicot-common hexaploidy (ECH) ancestor, initially revealed from the *Arabidopsis* genome [12] and later detailed using the grape genome [13, 14]. After polyploidization, a genome may often be unstable and subjected to extensive fractionation, with loss of many genes, rearrangement of chromosomal segments, and reduction in chromosome numbers, eventually producing a highly complex genome with interweaving intra-genomic homology [7–10].

These sequential paleopolyploidization events make it difficult to not only deconvolute their genome structure but also determine their composition and function. Obviously, insufficient analyses resulted in incorrect interpretations of the structure, evolution, and/or functional innovation of whole genomes and key gene families [15–18]. We recently developed a pipeline involving homologous gene dotplotting and characterizing event-related gene collinearity to help the analysis of complex genomes. The implementation of this pipeline with Cucurbitaceae genomes revealed an overlooked paleotetraploidization events that occurred ~ 100 million years ago (Mya), which may have contributed to the establishment and fast divergence of the entire Cucurbitaceae family [19].

Here, using the well-characterized grape (*V. vinifera*) and coffee (*C. canephora*) genomes as references, which are relatively simple genomes and are likely not affected by any polyploidization event after the ECH, we have reanalysed the carrot genome. We managed to infer the scale, nature, and time of polyploidization events. With the developed pipeline, we produced an alignment of collinearity-supported paralogous and orthologous genes that are related to each of the polyploidization and speciation events. A deep analysis indicated that several rounds of polyploidy events contributed to the expansion of gene families responsible for carotenoid accumulation and shaping underlying regulatory pathways in the carrot genome.

## Results

### Homologous gene collinearity

We inferred collinear genes within each genome and between carrot and coffee or grape reference genomes using ColinearScan [20], which provides a function for evaluating the statistical significance of blocks of collinear genes **(**Additional file 2**:** Tables S1 and S2). For the blocks with four or more collinear genes, we found the highest number of duplicated genes in carrot (1192–7142 pairs) and the fewest in grape (111–1831 pairs), while coffee contained 408–2436 **(**Additional file 2**:** Table S1). The carrot genome also retained the longest collinear fragments (122 gene pairs) compared with grape (61 gene pairs) or coffee (95 gene pairs). This indicated that carrot has a more complex and collinear genome.

With respect to intergenomic homology, there were 15,712–20,939 collinear gene pairs between the three genomes **(**Additional file 2**:** Table S1). For the blocks with four or more collinear genes, the number of collinear genes between grape and carrot was higher and the collinear blocks were shorter than those between grape and coffee. For blocks with > 50 collinear genes, there were 34 grape-carrot blocks (average 74.94 collinear genes) compared to 56 grape-coffee blocks (average of 112.95 collinear genes). The blocks between carrot and coffee genomes were better preserved than those between carrot and grape genomes. These findings could be explained by the occurrence of additional polyploidization events in the carrot genome, which likely resulted in greater genome fractionation **(**Additional file 2**:** Tables S1 and S2).

### Evidence of two paleotetraploidization events in *Daucus c. carota*

**Fig. 1:**
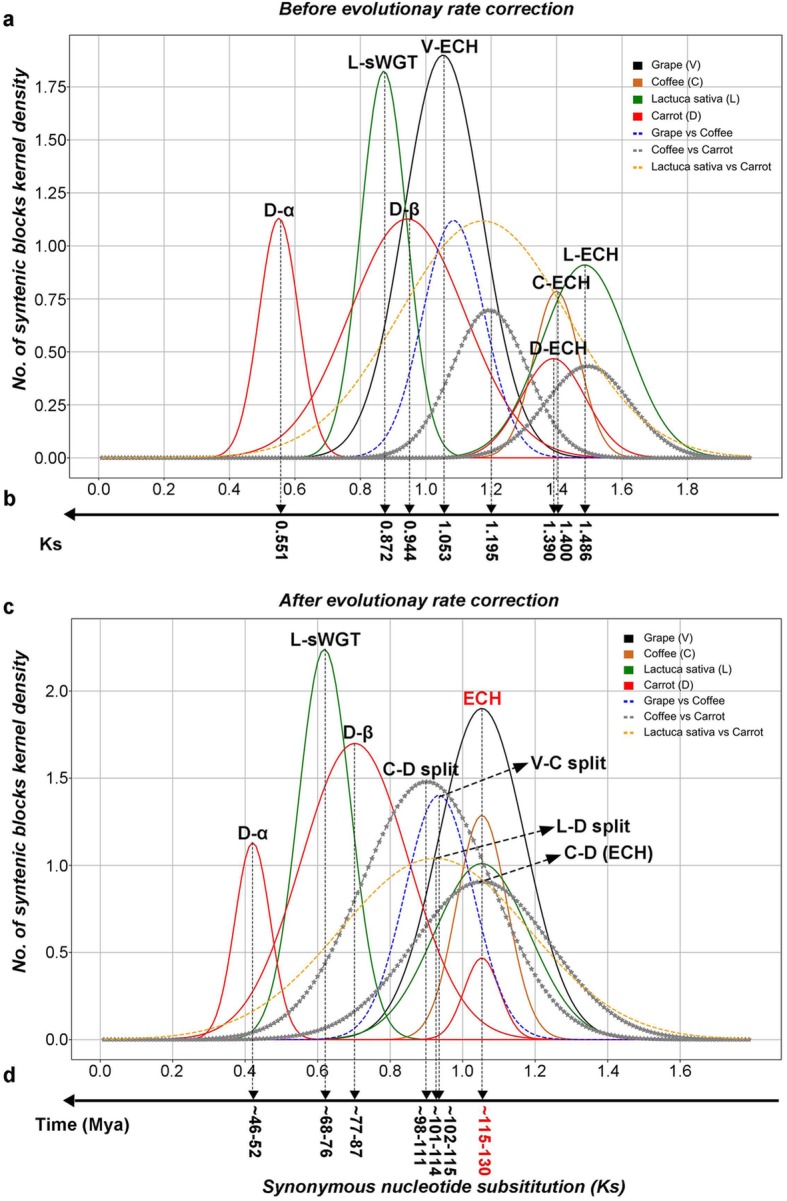
Original and corrected synonymous nucleotide substitutions between collinear genes (Ks). Dc-α, recent tetraploidization; Dc-β, Apiales-common tetraploidization; ECH, core-eudicot-common hexaploidization;. Continuous curves show Ks distribution within a genome, and broken curves show Ks distribution between genomes. **a** Distributions fitted using original Ks values; **b** inferred means; c distributions fitted using corrected Ks values; **d** inferred evolutionary dates

Using the collinear gene pairs inferred above, we estimated the synonymous substitution divergence (Ks) between each collinear gene pair. The Ks distribution in carrots had a clear tri-modal structure, peaking at 0.551 (+/− 0.06), 0.944 (+/− 0.176), and 1.390 (+/− 0.099) **(**Fig. 1a**)**; this result indicates three large-scale genomic duplication events, likely polyploidization events, corresponding to the events previously named Dc-α, Dc-β, and ECH, respectively.

Using homologous gene dotplots, we screened blocks with the median Ks of each block between every two genomes and managed to locate homologous correspondence to distinguish orthologous regions, which were established due to the split between plants, and outparalogous regions, which were established due to shared polyploidization events (Additional file 1**:** Figures. S1–3). In the grape-carrot dotplot, the 19 grape chromosomes were shown in seven colours, corresponding to seven ancestral eudicot chromosomes before the ECH, each having three homologous regions in the extant grape genome [13, 14]. For one carrot chromosome region in the grape-carrot dotplot **(**Additional file 1**:** Figure. S2), an orthologous grape chromosomal region was inferred due to its better DNA similarity (more collinear genes and a smaller median Ks) compared to its outparalogous regions in grape, the latter was related to the ECH. Often, these measures consistent inference to distinguish orthologous blocks from outparalogous ones. Therefore, we outlined orthologous regions using rectangles with solid and dashed lines to distinguish different sources from the two extra duplication events **(**Additional file 1**:** Figures. S2 and S3). In certain outparalogous regions with little trace of collinear genes, due to widespread and complementary gene losses [21], homology between grape chromosomes and/or between grape and carrot can be used to transitively indicate actual homology among the outparalogous regions. The analysis in the coffee-carrot dotplot reinforced our inferences from grape and carrot **(**Additional file 1**:** Figure. S3).

**Fig. 2:**
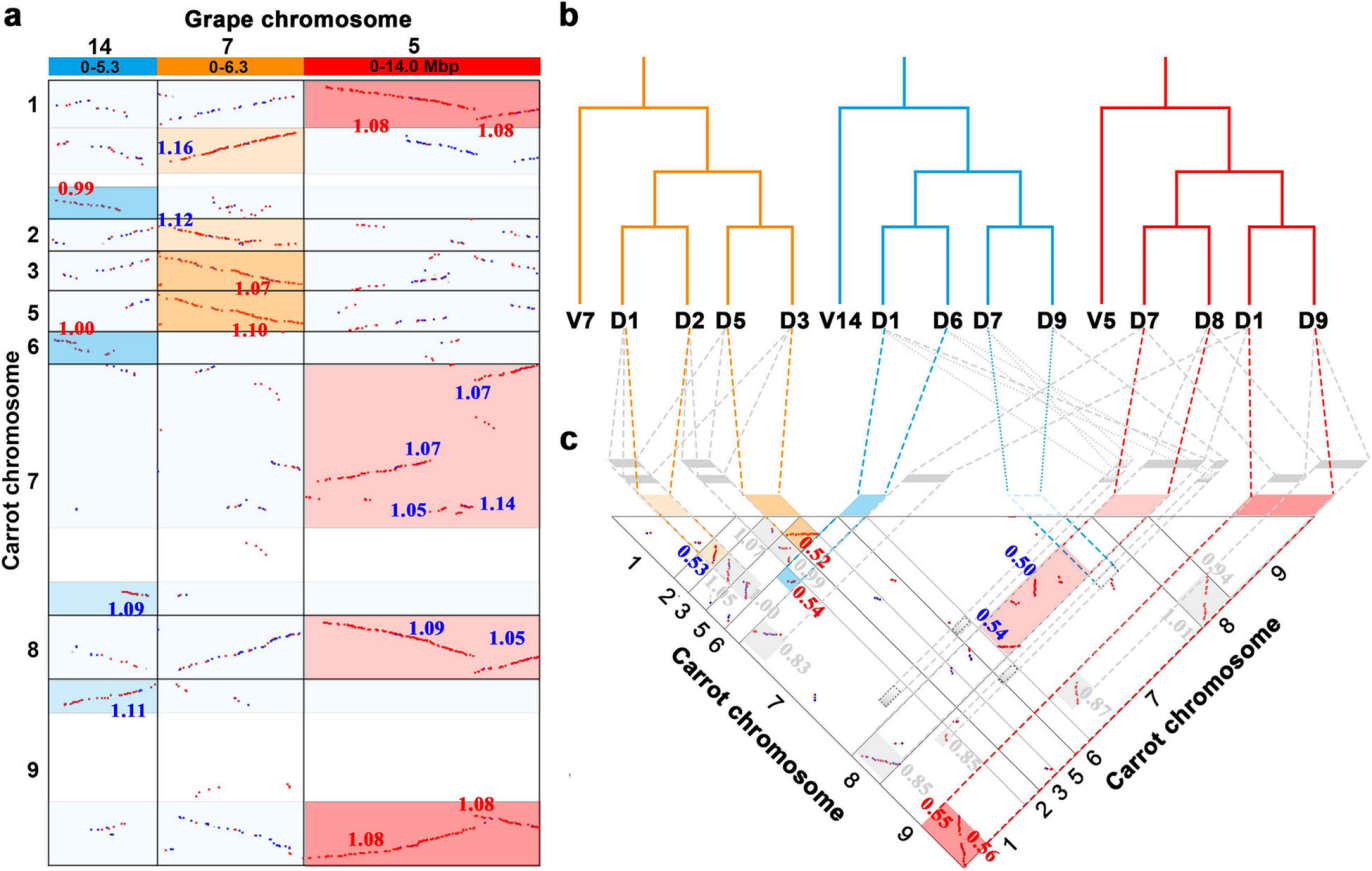
Examples of homologous gene dotplots between carrot and grape. Carrot and grape chromosome numbers are shown. Best-hit genes are represented by red dots, secondary hits as blue dots, and the other as grey dots. **a** Best-matched or orthologous copies between grape and carrot chromosomes. **b** Paralogous regions in carrot chromosomes (D1 to D9) corresponding to three grape chromosome regions (V5, V7, and V14). **c** Blocks showing gene collinearity. The numbers on collinear regions are the median Ks

If there had been an extra hexaploidization and tetraploidization event in carrot, as Iorizzo et al. reported [10], assuming no DNA loss, we would expect a grape gene (or chromosomal region) to have six best-matched or orthologous carrot genes (chromosomal regions) and 12 outparalogous genes (chromosomal regions). Here, our findings reveal, as an example, that Vv5, Vv7, and a large segment of Vv14 are a paralogous triplet produced by the ECH (we use Vv to denote the chromosomes of grape (*Vitis vinifera*), and Dc to denote the chromosomes of carrot (*Daucus carota*)). We found that Vv5 has four best-matched or orthologous copies in carrot chromosomes 1, 7, 8, and 9 (Fig. 2a**)**. The blocks circled by red rectangles contain 140, 190, 258, and 155 collinear genes for chromosomes 1, 7, 8, and 9, respectively. The median Ks of each block in these four best-matched regions are approximately 1.085, corresponding to the divergence of the grape-carrot ancestor. Orthologous regions of Vv5 in carrot are each outparalogous to the chromosome segments from Vv7 and Vv14, and the expected blocks are highlighted in Fig. 2a by light-blue rectangles. Many fewer collinear genes could be found in other outparalogous blocks (Vv7-Dc1, 42 collinear genes; Vv14-Dc1, 18; Vv7-Dc7, 57; Vv14-Dc7, 57; Vv7-Dc8, 70; Vv14-Dc8, 62; Vv7-Dc9, 60; Vv14-Dc9, 48).

Correspondingly, as to the positional information of orthology was revealed by the grape-carrot dotplot, we identified the paralogous regions in carrot. The paralogous regions in carrot chromosomes 1, 9 and 7, 8 were divided into two groups (Fig. 2b). The blocks in each group circled by red (between chromosomes 1 and 9) and light-red (between chromosomes 7 and 8) rectangles contain 120 and 256 collinear genes, respectively. The median Ks of these blocks were approximately 0.551, corresponding to the relatively recent tetraploidization (named Dc-α) (Fig. 2c). Four blocks between two groups circled by grey rectangles contain 46 (Dc1-Dc7), 88 (Dc1-Dc8), 66 (Dc7-Dc9), and 115 (Dc8-Dc9) collinear genes. The median Ks of these blocks were approximately 0.944, corresponding to the more ancient tetraploidization event (named Dc-β). Due to gene loss or translocation, some blocks are not on the expected chromosome regions, denoted by rectangles circled with grey dotted lines (Fig. 2c).

**Fig. 3:**
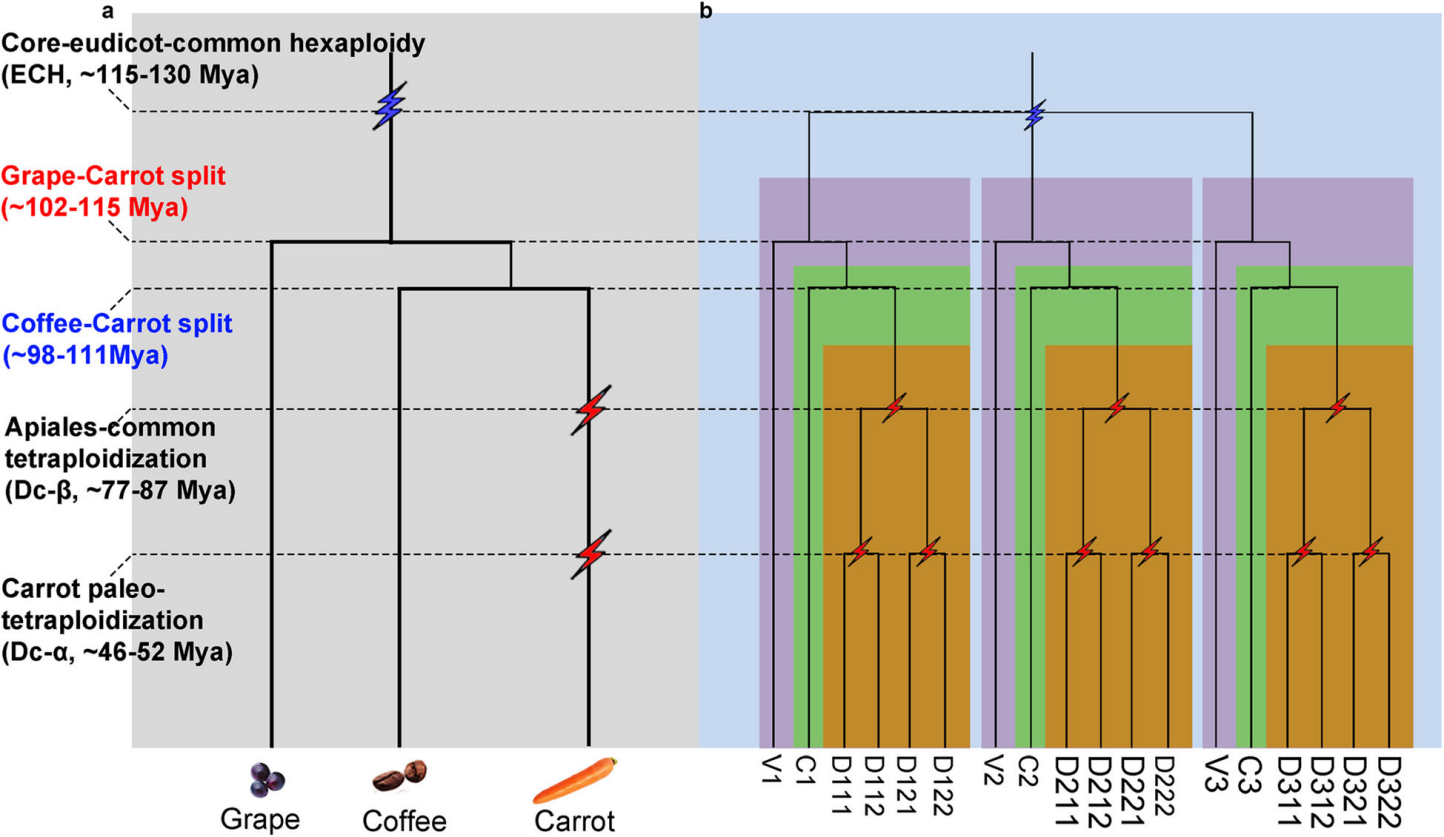
Species and gene phylogenetic trees for the carrot, coffee and grape genomes. a Phylogenetic tree of carrot (D), coffee (C), and grape (V): ECH is denoted by blue lightning bolts, and the two carrot paleotetraploidization events are denoted by red lightning bolts. b Gene phylogeny: three paralogous genes in the grape and coffee genomes are denoted by V1, V2, V3 and C1, C2, C3, produced by the ECH, and each has four orthologs and eight outparalogs in the carrot genome. For example, V1 has four orthologs D11, D12, D13, and D14 and eight outparalogs D21, D22, D23, D24, D31, D32, D33, and D34 in carrot. The species tree was produced based on our present analysis of homologous genes

Using a similar strategy for Vv7, orthologous regions and genes in carrot were identified, the homology (paralogy) between chromosomes 3 and 5 and between chromosomes 1 and 2 was produced by Dc-α, while the homology between the above two groups was produced by Dc-β (Fig. 2a-c). For the Vv14 segment, the corresponding orthologous regions and genes produced by Dc-α were also identified in two groups, those in chromosomes 1 and 6 and those in chromosomes 7 and 9, as a combinational result by Dc-β and Dc-α (Fig. 2a-c). Eventually, we identified the respective orthologous regions in carrot; grape paralogous chromosomes had different orthologous regions, and each had four best-matched copies (Fig. 2a). The corresponding orthologous regions in carrot were often broken into smaller regions and were even not present due to gene loss and chromosomal rearrangements after polyploidization. Fortunately, the duplication that resulted in similar break points, directions, and patterns of broken segments allowed us to infer that they were derived from the same ancestral chromosome or the same duplication event. One carrot chromosome region often corresponds to one best match and to two secondary matches of chromosome regions (Fig. 2c). From the coffee-carrot homologous gene dotplot, we found that for a large segment in coffee chromosome 3, there were four best matches in the carrot genome **(**Additional file 1**:** Figure. S4). The four best-matched regions were in carrot chromosomes 1, 8 and 7, 9, which represents the strongest evidence for the two paleotetraploidization events in carrot. In addition to the above example of tripled grape and coffee chromosomes, all other grape and coffee chromosomes similarly showed two sets of four best-matched carrot chromosomal regions **(**Additional file 1**:** Figures. S2 and 3), which strongly supported the notion of two paleotetraploidizations in carrot after the split from grape, coffee, and other eudicots **(**Fig. 3**)**.

We also performed gene phylogeny analysis to gain additional evidence in support of the two paleotetraploidization events in carrot. For 371 filtered groups of grape genes with at least three orthologous carrot genes, we constructed gene trees for 275 (74.12%) homologous gene groups; these showed the expected topology, which were in accordance with the two paleotetraploidization events in carrot. As expected, one grape gene had four of the best carrot orthologous genes divided into two groups, likely because of the two paleotetraploidization events. As such, a large number of groups have a topology supporting the two paleotetraploidization events **(**Additional file 1**:** Figure. S5).

### Event-related genomic homology

Inter- and intra-genomic comparisons helped to reveal the structural complexity of the carrot genome. Orthologous and paralogous genes were identified from speciation and polyploidy events. Detailed information on orthologous and outparalogous regions obtained from the dotplots **(**Additional file 2**:** Tables S3 and S4) was used to locate the orthologous and outparalogous genes (Additional file 2: Table S5–7). The analysis helped separate the duplicated genes from a genome into two ECH-related paralogs: the Dc-β-related paralogs, and the Dc-α-related paralogs. The ECH event produced 2424 paralogous pairs containing 3866 genes in 86 collinear regions in grape. In coffee, 1640 paralogous genes were found, containing 2768 genes in 92 collinear regions. In carrot, there were 5511 paralogous genes containing 6777 genes in 224 collinear regions. The two special paleotetraploidization events in carrot produced more paralogous regions, which was more than twice the number of those in grape. In theory, it should be fourfold as many as in grape without regard to loss. Notably, the numbers of genes showed more significant decreases than expected. For the ECH-related carrot genes (658 genes), the number was much smaller than in grape (3866) or coffee (2050), which was very likely due to the instability of the carrot genome after the extra two paleotetraploidization events **(**Table 1**).**

**Table 1:** Number of duplicated genes within selected genomes related to ECH, Dc-β, and Dc-α

Species	^a^ECH-related	^b^Dc-β-related	^c^Dc-α-related
*V. vinifera* (grape*)*	^d^ 86/2424/3866	–	–
*C. canephora* (coffee)	^d^ 54/1154/2050	–	–
*D. carota* (carrot)	^d^ 53/366/658	111/1105/2018	68/828/1533

^a^Core eudicot-common hexaploidy (ECH); ^b^ Apiales-common tetraploidization; ^c^ recent paleotetraploidization; ^d^ numbers of blocks/gene pairs/gene numbers

As expected, gene collinearity revealed better intergenomic than intragenomic homology. For example, 10,907 (35.48%) carrot genes had coffee orthologs, 5480 (17.83%) had coffee outparalogs, 9096 (29.59%) carrot genes had grape orthologs, and 4324 (14.07%) had grape outparalogs. Similar findings appear in the grape and coffee alignment, and additional information can be found in Additional file 2**:** Table S5–7.

### Multiple genome alignment

Using the grape genome as a reference and filling collinear gene IDs into a table, we constructed hierarchical and event-related multiple-genome alignments, producing a table of homologous genes [14] **(**Additional file 1**:** Figure. S5, Additional file 3: Table S8). This homologous collinear table was used to store inter- and intra-genomic homology information and to reflect three polyploidization events and all salient speciation. To accommodate genes specific to carrot, specifically those not available in the grape genome or those not represented by the above alignment table, we also constructed a genomic homology table with coffee as a reference **(**Additional file 1**:** Figure. S6, Additional file 3: Table S9), which supported the paleotetraploidization evidence in carrot and better represented carrot gene collinearity.

### Evolutionary dating of polyploidization events

By calculating synonymous substitutions (Ks) on synonymous nucleotide sites within grape, coffee and carrot and between them, we have successfully estimated the times of the sequential paleotetraploidization events Dc-β, Dc-α, and other key events. The different polyploidization events produced paralogs might overlap distributions but are abnormal for having long tails, especially in the large value sites, so we adopted an effective approach to find the major normal distributions in the observed Ks distribution (for more details can see Wang et al. 2018) [19, 22]. Therefore, the locations of the peaks and their variances were determined statistically **(**Fig. 1a**,** Additional file 2**:** Table S10). The ECH-related Ks peaks from the different genomes analysed were substantially different, with that of grape at Ks = 1.053 (+/− 0.120), coffee at Ks = 1.400 (+/− 0.070), carrot at Ks = 1.390 (+/− 0.099), and lettuce at Ks = 1.486 (+/− 0.060). These values suggest that the evolution rate of grape was the slowest among them, and the evolution rate of coffee, carrot, and lettuce was faster than that of grape by 32.95, 32.00, and 41.12%, respectively.

Significant differences in evolutionary rates lead to distortion when inferring occurrence times of evolutionary events. Here, based on an improved version of an approach that we previously developed [15, 23–27], we performed evolutionary rate correction by aligning the peaks of the ECH event to the same location (see Methods for details) **(**Fig. 1b**,** Additional file 2**:** Table S11). This correction aligned the ECH peaks to the same location, showing that it could correct the rate differences that accumulated after the ECH event between carrot and grape. Supposing that the ECH event occurred ~ 115–130 Mya [13, 28], adopted by previous publications [14, 29, 30], we inferred that the Dc-β and Dc-α events occurred ~ 77–87 Mya and ~ 46–52 Mya, respectively. Meanwhile, we found that Dc-β occurred in the Apiales (representative genome carrot) lineage after their split from Asterales (lettuce) ~ 98–111 Mya [4] and likely also after the Apiales-Bruniales divergence ~ 86.8 Mya [4], possibly playing an important role in the establishment of Apiales plants.

Homologous gene dotplotting provided further evidence that Dc-β was in the Apiales lineage but not in the Asterales lineage. Comparing the grape and lettuce genomes, we found that a grape gene (or chromosomal region) had three best-matched lettuce genes (chromosomal regions) **(**Additional file 1**:** Figure. S7). This indicated that a whole-genome triplication rather than a whole-genome duplication event occurred after the ECH, the basal genome of the Asterales including lettuce. By constructing homologous gene dotplots **(**Additional file 1**:** Figure. S8), we found that a lettuce chromosomal region had four best-matched (or orthologous) carrot chromosomal regions and often eight outparalogous chromosomal regions; a carrot chromosomal region had three best-matched (or orthologous) lettuce regions and six outparalogous regions. This supports two tetraploidization events in the carrot lineage and one hexaploidization event in the lettuce lineage.

### Genomic fractionation

**Fig. 4:**
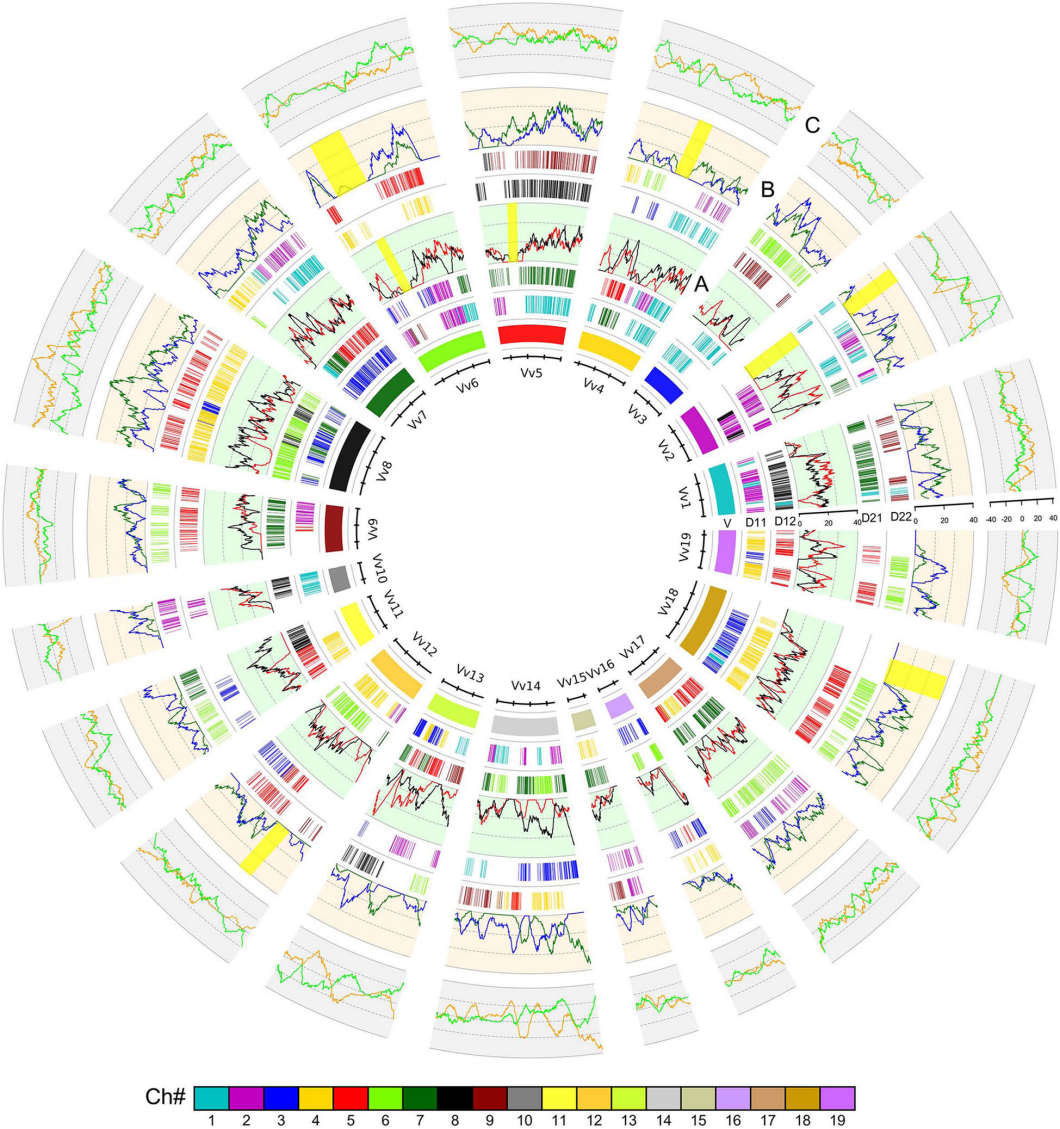
Homologous alignments and carrot subgenome gene retention along corresponding orthologous grape chromosomes. Genomic paralogy and orthology information within and among genomes is displayed in five circles. The short lines forming the innermost barrel medic chromosome circles represent predicted genes. Each of the barrel medic and grape paralogous chromosomal regions has four orthologous copies in carrot. Each circle is formed by short vertical lines that denote homologous genes, which are coloured to indicate chromosome number in their respective source plant as shown in the colour scheme at the bottom. **a** Rates of retained genes in sliding windows of carrot homologous region group 1 (red) and homologous region group 2 (black); **b** rates of retained genes in sliding windows of carrot homologous region group 3 (green) and homologous region group 4 (blue); **c** differences between groups 1 and 2 (orange yellow) and groups 3 and 4 (lime) are displayed. Large patches of chromosomal segmental losses (yellow)

**Fig. 5:**
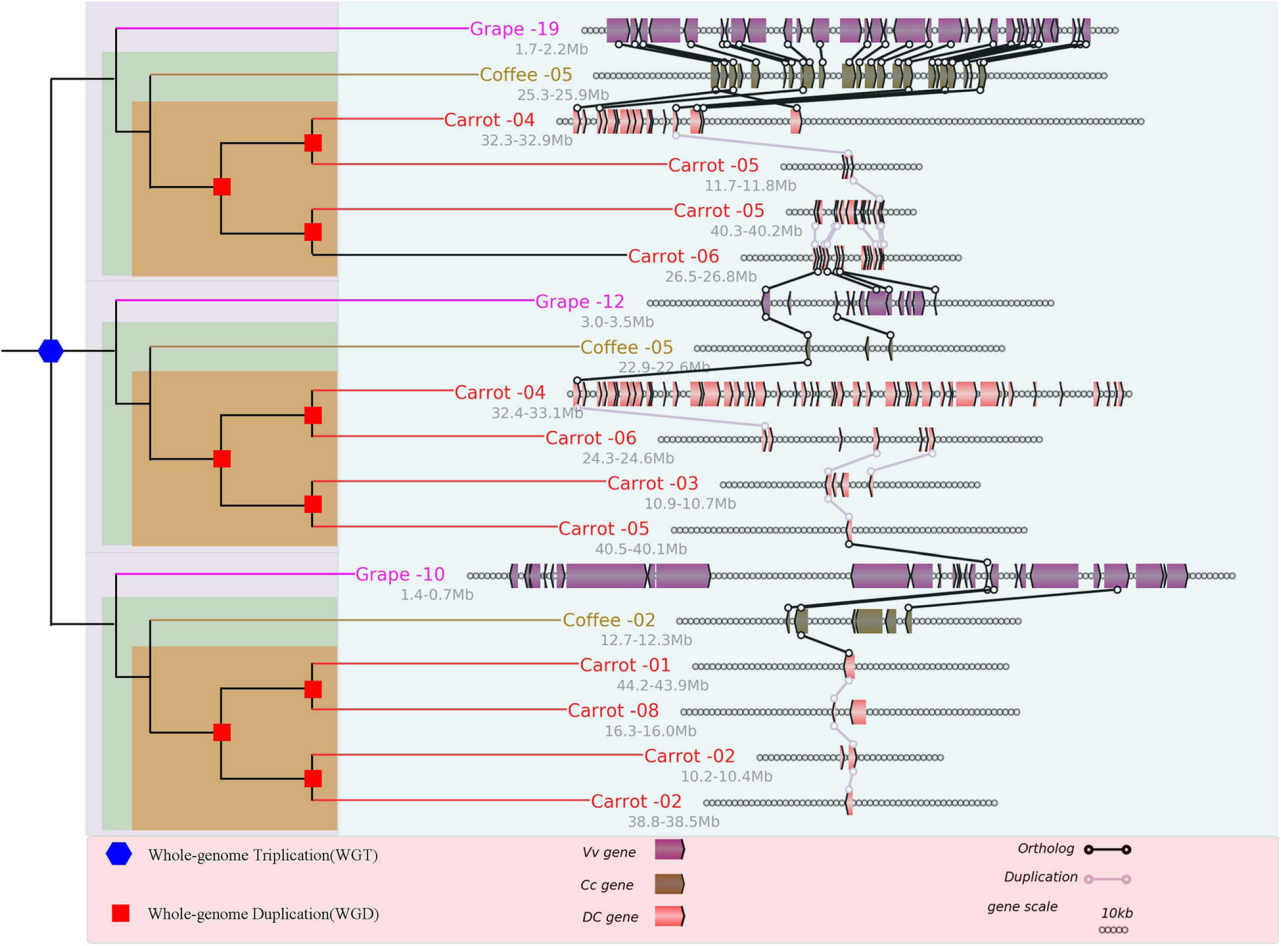
Local alignment of the carrot genome with the grape genome as a reference. Details of a short segment of alignment, chosen from the global alignment in Fig. 4. Homologous block phylogeny (left): three paralogous chromosome segments in the grape genome, Grape-10, Grape-12, and Grape-19, derived from ancestral chromosomes affected by ECH, each with four orthologous carrot chromosome segments. Genes are shown by rectangles. Homologous genes between neighbouring chromosomal regions are linked with lines

A large number of gene losses and translocations have occurred after genome duplications in carrots. Intragenomic gene collinearity analysis in carrot indicated that a tiny fraction (0.1%, 25 regions) preserved eight copies of duplicates, likely produced by three recursive polyploidy events, which should exist as 12 copies if preserving perfect gene collinearity (Additional file 2**:** Table S12). Intergenomic analysis with grape as a reference revealed 0.3% (63) preserved copies in carrot duplicated regions (Additional file 2**:** Table S13). We then calculated gene retention or removal rates per referenced chromosome **(**Figs. 4-5**,** Additional file 1: Figure. S9). Grape and coffee as a reference both showed much lower collinear gene correspondence with carrot. Different grape chromosomes had collinear gene loss rates of 71–92% in each of their four sets of orthologous regions (Additional file 2**:** Table S14). Approximately 71, 79, 86, and 82% of genes on grape chromosome 2 did not have collinear genes in one of the four sets of carrot orthologous regions, and 66% of genes did not have correspondence in all homologous regions. Different coffee chromosomes had collinear gene loss rates of 54–89% in each of their four sets of orthologous regions (Additional file 2**:** Table S15). Similarly, 78, 86, 71, and 83% of genes on coffee chromosome 8 did not have collinear genes in one of the four sets of carrot orthologous regions, and 61% of genes did not have correspondence in all homologous regions. Between two sets of the same polyploidization paralogous regions, different grape (coffee) chromosome gene loss rates were not all similar 0–0.1 (0–0.29). Grossly, these findings show extensive gene deletions or relocations after polyploidization events.

To explore the mechanism underlying genomic fractionation, we characterized the runs of continual gene removal in carrot compared to the other referenced genomes [31] (methods detailed by Wang et al. 2015a). Although there were patches of chromosomal segments removed (likely segmental loss) **(**Additional file 1**:** Figures. S5 and S6), most of the runs of gene deletions were 15 continual genes or fewer. A statistical fitness regression showed a deletion pattern following a near geometric distribution **(**Additional file 1**:** Figure. S10, Additional file 2: Table S16). With grape and coffee genomes as references, carrot had a gene removal pattern following the geometric distribution (geometric parameter *p* = 0.221–0.249, the probability of removing one gene at a time, and goodness of *p*-value = 0.93 in fitting F-test to accept the fitness). This shows that 38–42% of genes were removed in runs containing 1 or 2 genes, indicating a mechanism of fractionation of short DNA segment removal, or approximately 5–10 kb DNA in length. It seems that short removal runs accounted for the majority initially and then recursive removals overlapping previous ones elongated the observed length of runs.

Furthermore, we calculated the retention level with 100 genes and steps of one gene as a sliding window **(**Additional file 4**:** Table S17). Homologous regions produced by Dc-α were grouped in subgenomes A11-A12 and A21-A22 (A means an inferred subgenome); meanwhile, A11-A21, A11-A22, A12-A21, and A12-A22 were related to Dc-β. Using the grape genome as a reference, for Dc-α, there were only 25.48 and 22.01% homologous sliding windows for A11-A12 and A21-A22, respectively, showing no significant difference (less than 5% difference in gene retention rates: *p* < 0.05) in gene removal. At the same time, for Dc-β, there were only 22.01, 27.41, 25.87, and 19.69% homologous sliding windows for A11-A21, A11-A22, A12-A21, and A12-A22, respectively, showing no significant difference (p < 0.05) in gene removal. Often, diverged gene retention rates between subgenomes produced by two duplication events indicate the likely allotetraploidization nature for both Dc-α and Dc-β. For further determination, we used coffee as a reference genome to calculate the retention and found stronger evidence **(**Additional file 4**:** Table S18). For Dc-α, there were only 82.6 and 90.36% homologous sliding windows for A11-A12 and A21-A22, respectively, showing significant differences (p < 0.05) in gene loss. For Dc-β, there were only 76.89–81.7% homologous sliding windows showing significant differences (p < 0.05) in gene retention. These findings support the hypothesized allotetraploidization nature of the two events.

**Fig. 6:**
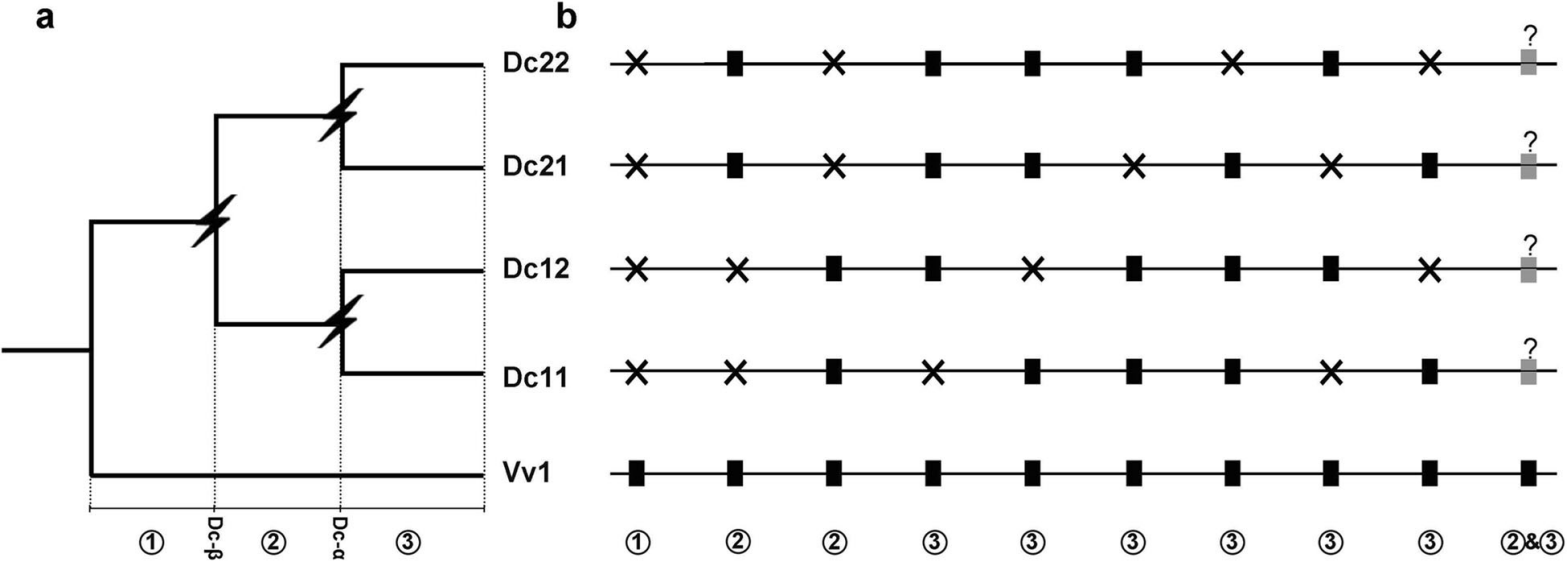
Detection of gene loss in carrots during different evolutionary periods. “?” represents only one gene that may exist. a Gene phylogeny: If no gene loss occurred, a grape gene is anticipated to have four carrot orthologs, Dc11, Dc12, Dc21, and Dc22. Two carrot paleotetraploidization events were denoted by lightning bolts. b Rectangles show gene; ‘ × ’ shows gene loss; alternative situations of the carrot gene loss are divided into three categories: ① carrot genes loss before Dc-β; ② carrot genes loss between Dc-β and Dc-α; ③ carrot genes loss after Dc-α

With grape as a reference, we checked gene loss in carrot based on the homologous alignment table (Fig. 6**)**. According to alternative erosion of gene collinearity, gene losses in carrots can be classified into three categories: 1, carrot gene loss before Dc-β; 2, carrot gene loss between Dc-β and Dc-α; and 3, carrot gene loss after Dc-α. We inferred that 1330, 5594, and 6312 carrot genes were lost before Dc-β, between the occurrences of Dc-β and Dc-α, and after the occurrence of Dc-α, respectively. This inference suggested that widespread genes were lost after two recent polyploidization events, while before them, the ancestral genome had been relatively stable. Apparently, the different rates of gene loss among the three periods may have been influenced by two extra polyploidizations, which supports the idea that species with more rounds of polyploidization may suffer more gene loss. Furthermore, both the 84% ratio of gene loss after Dc-α and the 86–87% ratio of gene loss after Dc-β showed a large amount of gene loss after polyploidization; this was similar to the nearly 70% gene loss that occurred in the cotton genome after decaploidization and the approximately 69% gene loss within extant soybean, which was also affected by two extra tetraploidization events after the ECH [15, 25].

In this study, we found some genes with repeat DNA fragments corresponding to two or more homologous genes in grape or coffee. We found 9114 (out of 32,113) carrot genes with repetitive fragments in their formation. For example, the sequence of the gene DCAR_003216 (with the most repeat fragments amounting to 17) is the fusion of two grape tandem genes, Vv13g1246 and Vv13g1253. The sequence of the gene DCAR_003216 was nearly double that of the coffee gene Cf02_g28080. The above observation could be explained by the preservation of two ancient tandem genes in grape: their fusion in carrot, and the loss of one copy of the tandem genes in coffee.

### Polyploidization and carotenoid pathway genes

**Fig. 7:**
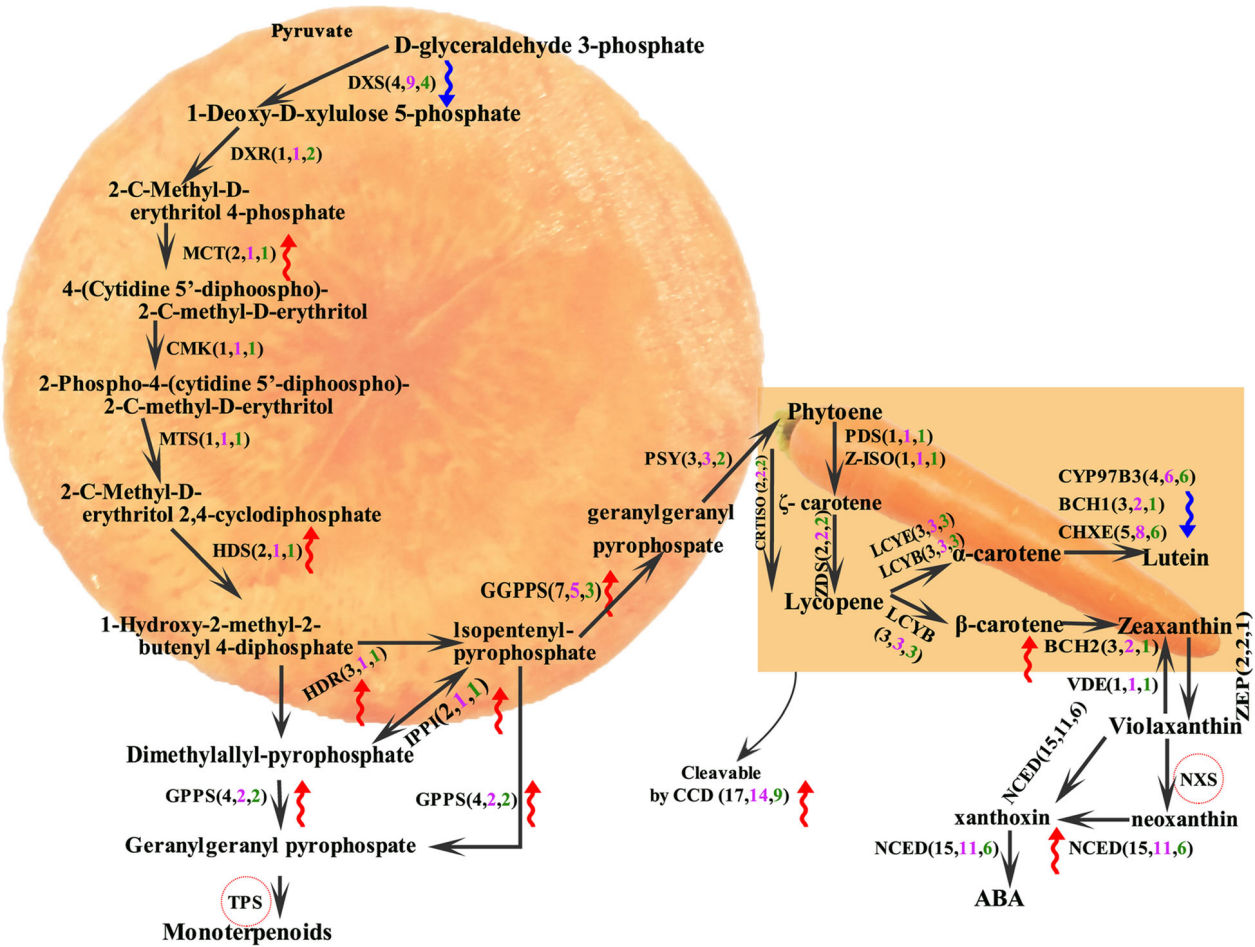
MEP and carotenoid pathways. Numbers in parentheses denote the genes in carrots (with black), grapes (with purple) and coffee (with green) in turn. Red and blue arrows indicate an increase or decrease in the number of copies in carrot compared to grape and coffee

In total, there were three identified polyploidization events in carrot (ECH, Dc-β and Dc-α events), and they contributed to the expansion of MEP pathways. Here, we detected gene homologs in the MEP and carotenoid pathways in carrot, grape, and coffee through BLASTP (E-value <1e-10, and score > 150) **(**Fig. 7**,** Additional file 2: Table S19) using the previously reported genes in the pathways as searching seeds [11]. In the MEP and carotenoid pathways of carrot, 28% of genes are related to the ECH event, while 96 and 92% are related to Dc-β and Dc-α, respectively. Compared with MEP pathway (only 4-(cytidine 5-phospho)-2-C-methyl-D-erithritol kinase (CMK) and 4-(cytidine 5-phospho)-2-C-methyl-D-erithritol kinase (MTS) had the same copy number in carrot, grape, and coffee genomes), the number of gene copies in the carotenoid pathway (15-cis-phytoene desaturase (PDS), ζ-carotene isomerase (Z-ISO), carotenoid isomerase (CRTISO), ζ-carotene desaturase (ZDS), lycopene ε-cyclase (LCYE) and violaxanthin de-epoxidase (VDE) had the same copy number in carrot, grape, and coffee genomes) is relatively stable. The gene with the highest copy number in carrot, grape, and coffee is the carotenoid cleavage dioxygenase (CCD) gene with 17, 14, and 9 copies, respectively, and the second is the 9-cis-epoxycarotenoid dioxygenase (NCED) gene (15, 11, 6 copies, respectively). Although both CCD and NCED play a negative role (also having geranyl diphosphate synthase (GPPS) and beta-carotene hydroxylase (BCH)) in carotenoid biosynthesis, the copy numbers of the genes 2-C-methyl-D-erythritol 4-phosphate cytidylyltransferase (MCT), 4-hydroxy-3-methylbut-2-en-1-yl diphosphate synthase (HDS), geranylgeranyl pyrophosphate synthase (GGPPS), 4-hydroxy-3-methylbut-2-enyl diphosphate reductase (HDR) and isopentenyl-diphosphate delta-isomerase I-like (IPPI) increased in carrot slightly; this led to the increase in carotene pathway precursor, which might be the key factors contributing to the increase in carotene content in carrots. The carotenoid pathway is relatively conservative in the three species, with the same number of copies, except for the BCH, cytochrome P450 97B3 and CHXE genes. The copy numbers of CYP97B3 and CHXE, which control the degradation of α-carotene, decreased, and BCH, which regulates degradation of β-carotene, increased in carrots; this may be a reason for why the levels of α-carotene are 10 times higher than those of β-carotene in carrot.

## Discussion

### Tetraploidization of dc-β instead of triplication

Plant genomes often have complex structures due to recursive polyploidization and genome repatterning events [32, 33], which increase the difficulty of deconvoluting genomic structures, understanding genome formation, or exploring the origin and functional evolution of genes, gene families, and pathways. A crucial consideration to decipher the genome structure after rounds of polyploidization is to distinguish orthologous from outparalogous colinear blocks in inter-genomic comparisons. Gene dotplots can be used to achieve this distinction and were previously used to infer three rounds of paleo-polyploidy in *Arabidopsis thaliana* [12]. This comparative genomics pipeline that we streamlined has been efficiently applied to genome structure analysis of several plant species or groups, such as cotton [15], durian [22], cultivated peanut [34], legume [25] and Cucurbitaceae [19]. In fact, a previous study inferred WGT (Dc-β) and WGD (Dc-α) based on the analysis of syntenic gene blocks (one grape region had 6 carrot blocks) [11], which might mix the orthologs and outparalogs. As indicated, when using grape and coffee as reference genomes, the analyses of the carrot genome revealed a 1:4 relationship, dividing the carrot paralogous regions into two groups. The 1:4 ratio indicated that the Dc-β event was a tetraploidization instead of a triplication, as reported previously [11]. The establishment of orthologous and paralogous gene lists, inferred for each polyploidization and/or speciation event, will constitute the Apiales comparative genomics platform to be used in future studies.

Furthermore, approximately 74.12% (275 in 371) of homologous gene topology trees support the two paleotetraploidization events in carrots, which is strong evidence. Regarding the grass-common tetraploidization event, 31–37% of homologous gene topology trees [7, 10] and 38.9% (68 in 175) of homologous gene topology trees supported the cucurbit-common tetraploidization [19]. The other homologous gene topology trees that did not meet expectations are likely caused by divergent evolutionary rates of recursively duplicated genes.

### Dc-α and dc-β were both likely allotetraploidizations

Ancient WGDs have played an essential role in plant adaptation to extreme environment, such as the Cretaceous–Paleocene (K-Pg) boundary, the polyploidy contributed more gene families related to darkness and cold stress [35]. Polyploids with unbalanced subgenomes (regarded as allopolyploids) established the major flora, as reported in maize [36], bread wheat [37], brassica [38] and Cucurbitaceae plant species [19]. The allopolyploids had a long time span, with some of them just occurred thousands years like canola and bread wheat, and others occurred tens of millions years maize and Cucurbitaceae. Sequential allopolyploids in carrot may confer genetic and environmental advantages that enhance survival.

### Scope of paleotetraploidization in carrot

By using collinear gene block analyses, we inferred that the Dc-β and Dc-α polyploidization events occurred ~ 77–87 Mya and ~ 46–52 Mya, respectively. The occurrence time of Dc-β was seemingly near the divergence time of carrot and lettuce, which, according to a previous report, had taken ~ 72 and 93 Mya, respectively [4, 11]. With collinear ortholog analyses, we estimated that the carrot-lettuce divergence occurred 98–111 Mya, indicating that carrot and lettuce do not share the tetraploidization events. In addition, the homologous dotplot of carrot and lettuce showed that the ratio of homologous regions in the two genomes was 4:3 (Additional file 1**:** Figure S8), meaning a whole-genome triplication in the lettuce lineage occurred. In summary, with the analyses presented here, we demonstrate that two tetraploidization events are specific to Apiales and may have led to the formation of the plant lineage.

### Possible factors of carotenoid-rich carrots

Polyploidizations have always contributed to the evolution of key traits, such as nodulation, NBS-LRR resistance, EIN3/EIL, cotton fibres, VC biosynthesis and recycling-associated genes [25, 30, 39, 40]. Based on the MEP and carotenoid pathway proposed by Iorizzo et al. [11], we analysed the association between regulatory genes and the different polyploidization events in the MEP and carotenoid pathway. We found that each polyploidy event affected the carotenoid accumulation pathway differentially. The Dc-β and Dc-α events contributed more than the ECH event in carrot, possibly because the Dc-β and Dc-α events occurred relatively recently, which might have promoted the formation of carrot. The changes in gene copy number in carrot, grape and coffee were compared horizontally, and some genes had the same copy number in three species. Interestingly, the copy number of CCD and NCED genes, genes related to carotenoid degradation, was higher in the carrot genome compared to the other reference genes, which contradicted the fact that carrot has a rich carotenoid content. The increased copy number of MCT, HDS, HDR, IPPI and GGPPS genes may have been a key factor for the actual carotenoid-enriched carrots.

### Evolutionary rates

The discrepancy of evolutionary rates among different species affects phylogenetic analysis and accurate time estimation. For example, cotton evolved 64% faster than durian [22], the coffee genome evolved 47.20% faster than the kiwifruit and grape genomes [39], and mulberry evolved much (even 3 times) faster than other Rosales species [41]. Here, we found that the evolutionary rate of grape was the slowest, while coffee, carrot and lettuce evolved faster than grape by 32.95, 32.00, and 41.12%, respectively. To perform authentic dating, the evolutionary rates of coffee and carrots were corrected by using grape with the slowest evolutionary rate.

## Conclusions

According to this study, hierarchical inference of homology revealed two tetraploidization events that shaped the carrot genome; these events likely contributed to the successful establishment of Apiales plants and the expansion of MEP pathway genes upstream of the carotenoid accumulation pathway.

## Methods

Genomic sequences and annotations were downloaded from the corresponding genome project website **(**Additional file 2: Table 20).

### Gene collinearity

Collinear genes were inferred using the ColinearScan algorithm and software [20]. The maximal collinearity gap length between genes was set as 50 genes as used previously [17, 23–25]. Homologous gene dotplots within a genome or between different genomes were produced using MCSCANX toolkits [42].

### Construction of the event-related collinear gene table

Using the grape genes as a reference, we constructed a polyploid event-related collinear gene table (Additional file 3**:** Table S8). The first column was filled with all grape genes, which were arranged in positions on chromosomes. Each grape gene may have two extra collinear genes for the ECH, so the grape genes filled other two columns. For the coffee genome, without extra duplications besides the ECH, we assigned one column close behind the grape columns. For the carrot genome, with the two paleotetraploidization events, we assigned four columns close behind the coffee columns. Therefore, the table had 18 columns, which reflected the homologous relationship among species after different polyploid events. For a grape gene, when there was a corresponding collinear gene in an expected location, the gene ID was filled in a cell of the corresponding column in the table. When it was missing, often due to gene loss or translocation in the genome, we filled in the cell with a dot. The coffee reference table was constructed similarly (Additional file 3: Table S9).

### Evolutionary tree construction with homologous collinear table

One grape gene had three or more orthologous carrot genes which were constructed evolutionary tree by using the maximal likelihood approach in PHYML [43] and the neighbour-joining approach in PHYLIP under default parameter settings [44].

### Nucleotide substitution

Synonymous nucleotide substitutions (K_S_) between homologous genes were estimated by running the BioPerl (Version: 1.007002) biostatistics package, Bio::SeqIO, Bio::Align::Utilities, Bio::Seq::EncodedSeq, Bio::AlignIO and Bio::Align::DNAStatistics, which implements the Nei–Gojobori approach [45].

### Evolutionary dating correction

To correct the evolutionary rates of ECH-produced duplicated genes, the maximum-likelihood estimates *μ* from inferred Ks means of ECH-produced duplicated genes were aligned to have the same values as those of grape, which had evolved the slowest. Supposing that a grape duplicated gene pair with a Ks value is a random variable distribution is X_G_~(*μ*_G_, *σ*_*G*_^2^), and for a duplicated gene pair in another genome, the Ks distribution is X_i_~(*μ*_i_, *σ*_*i*_^2^); we obtained the expectation of a relative difference in random variables with the following equation:

$$ \mathrm{r}=\left({\mu}_{\mathrm{i}}-{\mu}_G\right)/{\mu}_G. $$

To obtain the corrected X_i − correction_~(*μ*_i − correction_, *σ*_*i correction*_^2^), we defined the correction coefficient as follows:

$$ \frac{\mu_{\mathrm{i}-\mathrm{correction}}}{\mu_i}=\frac{\mu_{\mathrm{G}}}{\mu_i}={\lambda}_i, $$

and $$ {\mu}_{\mathrm{i}-\mathrm{correction}}=\frac{\mu_{\mathrm{G}}}{\mu_i}\times {\mu}_i=\frac{1}{1+r}\times {\mu}_i $$.

$$ {\lambda}_i=\frac{1}{1+r} $$

then,

$$ {\mathrm{X}}_{\mathrm{i}-\mathrm{correction}}\sim \left({\lambda}_{\mathrm{i}}{\mu}_i,{\lambda}_{\mathrm{i}}{\sigma_i}^2\right). $$

To calculate Ks of homologous gene pairs between two plants, *i*, *j*_,_ suppose that the Ks distribution is X_ij_ = (*μ*_*ij*_, *σ*_*ij*_^2^); we adopted the algebraic mean of the correction coefficients from two plants,

$$ {\lambda}_{\mathrm{i}\mathrm{j}}=\left({\lambda}_{\mathrm{i}}+{\lambda}_{\mathrm{j}}\right)/2, $$

then,

$$ {X}_{i- correction}\sim \left({\lambda}_{ij}{\mu}_{ij},{\lambda}_{ij}{\sigma_{ij}}^2\right). $$

Specifically, when one plant is grape, for the other plant, *i*, we have

$$ {X}_{iG- correction}\sim \left({\lambda}_i{\mu}_{iG},{\lambda}_i{\sigma_{iG}}^2\right). $$

## Supplementary information

**Additional file 1: Figure S1.** Homologous dotplot within carrot genome. **Figure S2.** Homologous dotplot between grape and carrot genomes. **Figure S3.** Homologous dotplot between coffee and carrot genomes. **Figure S4.** Examples of homologous gene dotplots between carrot and coffee. **Figure S5.** Trees with topology supporting the Dc-α and Dc-β.a-f. **Figure S6.** Alignment of carrot, coffee and grape genomes. **Figure S7.** Homologous dotplot between grape and lettuce. **Figure S8.** Homologous dotplot between carrot and lettuce. **Figure S9.** Homologous alignments and carrot subgenome gene retention along corresponding orthologous coffee chromosomes. Details in Fig. 4. **Figure S10.** Fitting a geometric distribution and gene loss rates in carrot as to the grape and coffee.

**Additional file 2: Table S1.** Number of homologous blocks and gene pairs within a genome or between genomes. **Table S2.** Number of homologous genes within a genome or between genomes. **Table S3.** Orthologous genomic regions between grape and carrot. **Table S4.** Orthologous genomic regions between coffee and carrot. **Table S5 S6. S7.** Paralogous, orthologous and out-paralogous gene pairs within a genome or between genomes. **Table S10.** Kernel function analysis of Ks distribution related to duplication events within each genome and between genomes (before evolutionary rate correction.) **Table S11.** Kernel function analysis of Ks distribution related to duplication events within each genome and between genomes (after evolutionary rate correction). **Table S12.** Homologous depth within carrot, coffee and grape genome. **Table S13.** Intergenomic homologous depth of carrotgenome with grape or coffee as reference. **Table S14.** Intergenomic homologous depth of carrotgenome with grape or coffee as reference. **Table S15.** Carrot gene loss rates and gene translocation with coffee as reference genome. **Table S16.** The observed distribution of gene loss and translocation numbers fitted by using different density curves of geometry distribution. **Table S19.** Carotenoid accumulation gene family. **Table S20.** Information of genomic data.

**Additional file 3: Table S8.** Homologous alignments of carrot with grape as reference. **Table S9.** Homologous alignments carrot genomes with coffee as reference.

**Additional file 4: Table S17.** Gene retention in homoeologous carrot regions as to grape genome. **Table S18.** Pindex of subgenome retention in plant genomes results.

## Data Availability

The data analysed during the current study originally downloaded from the JGI (https://phytozome.jgi.doe.gov/) and http://coffee-genome.org/. All data and materials generated or analyzed during this study are included in this article or are available from the corresponding author on reasonable request.

## Abbreviations

ECH: Core Eudicot-Common Hexaploidy
Mya: Million years ago
