# Supplementary figures and images for: Sequential Paleotetraploidization shaped the carrot genome
Source: BMC Plant Biol. 2020 Jan 31;20:52. doi: 10.1186/s12870-020-2235-7 (PMC6995200; doi:10.1186/s12870-020-2235-7)

# Carrot

1 2 3 4 5 6 7 8 9

Carrot

1

2

3

4

5

6

7

8

9

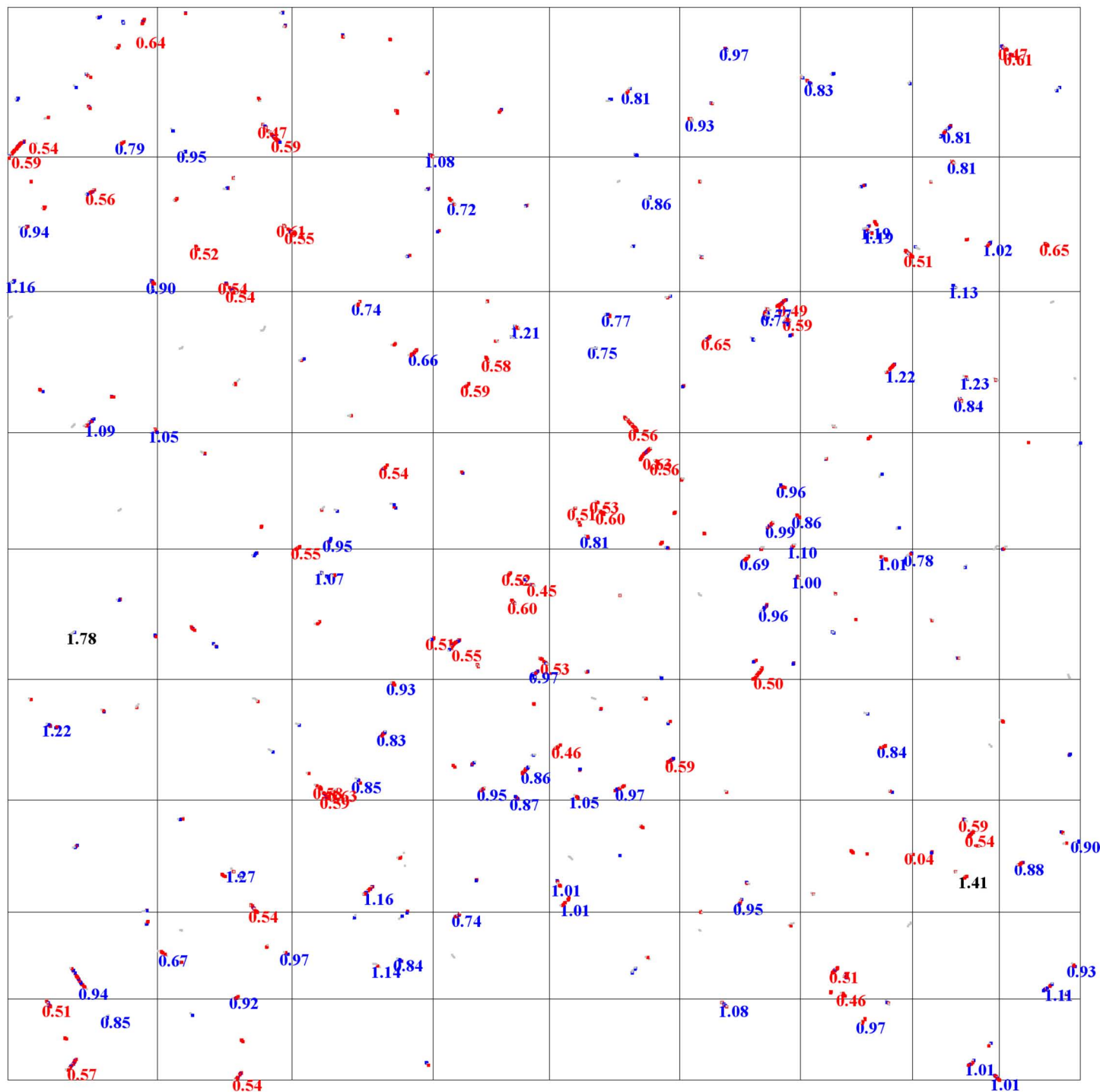

# Carrot

Grape

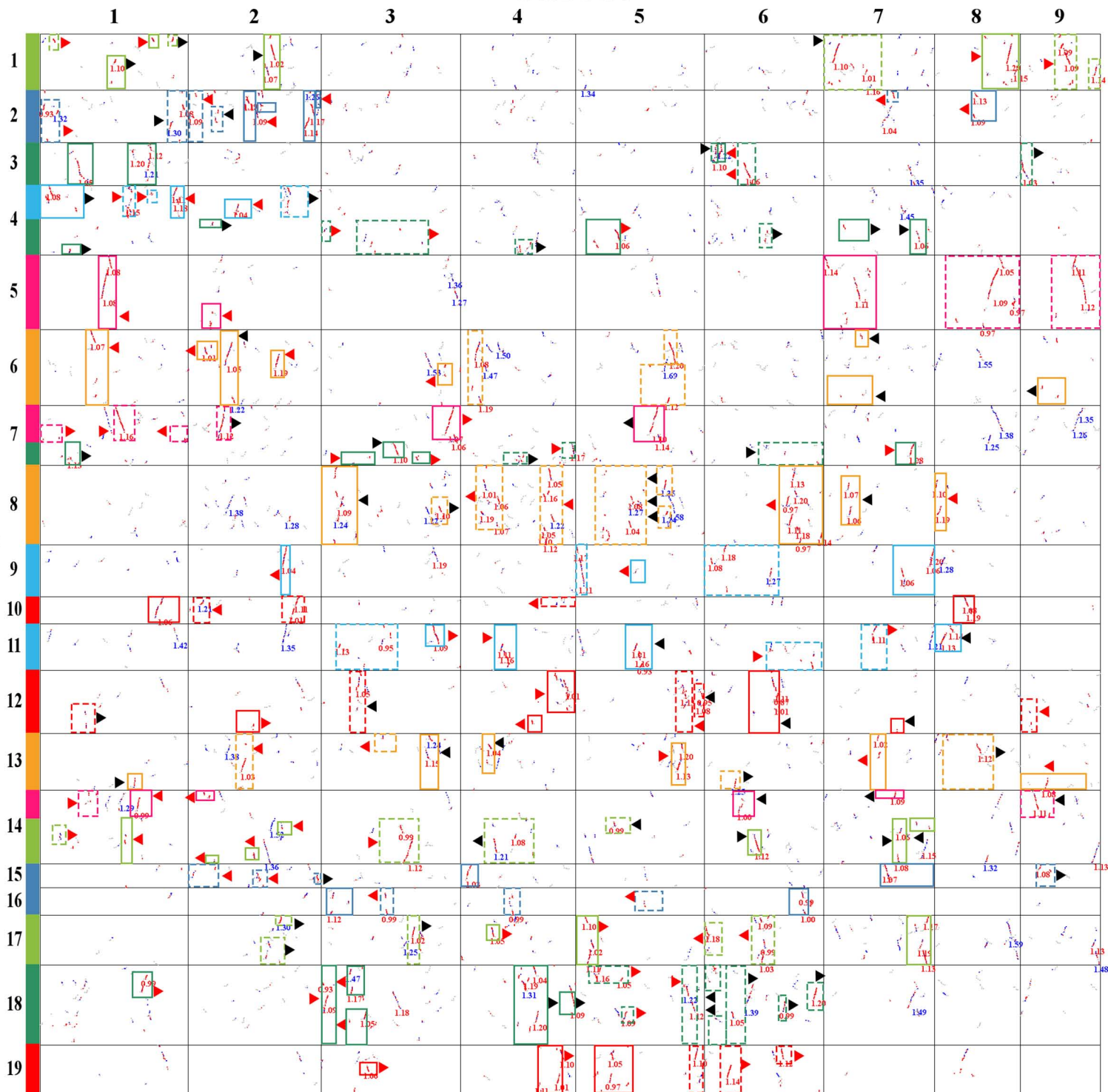

# Coffee

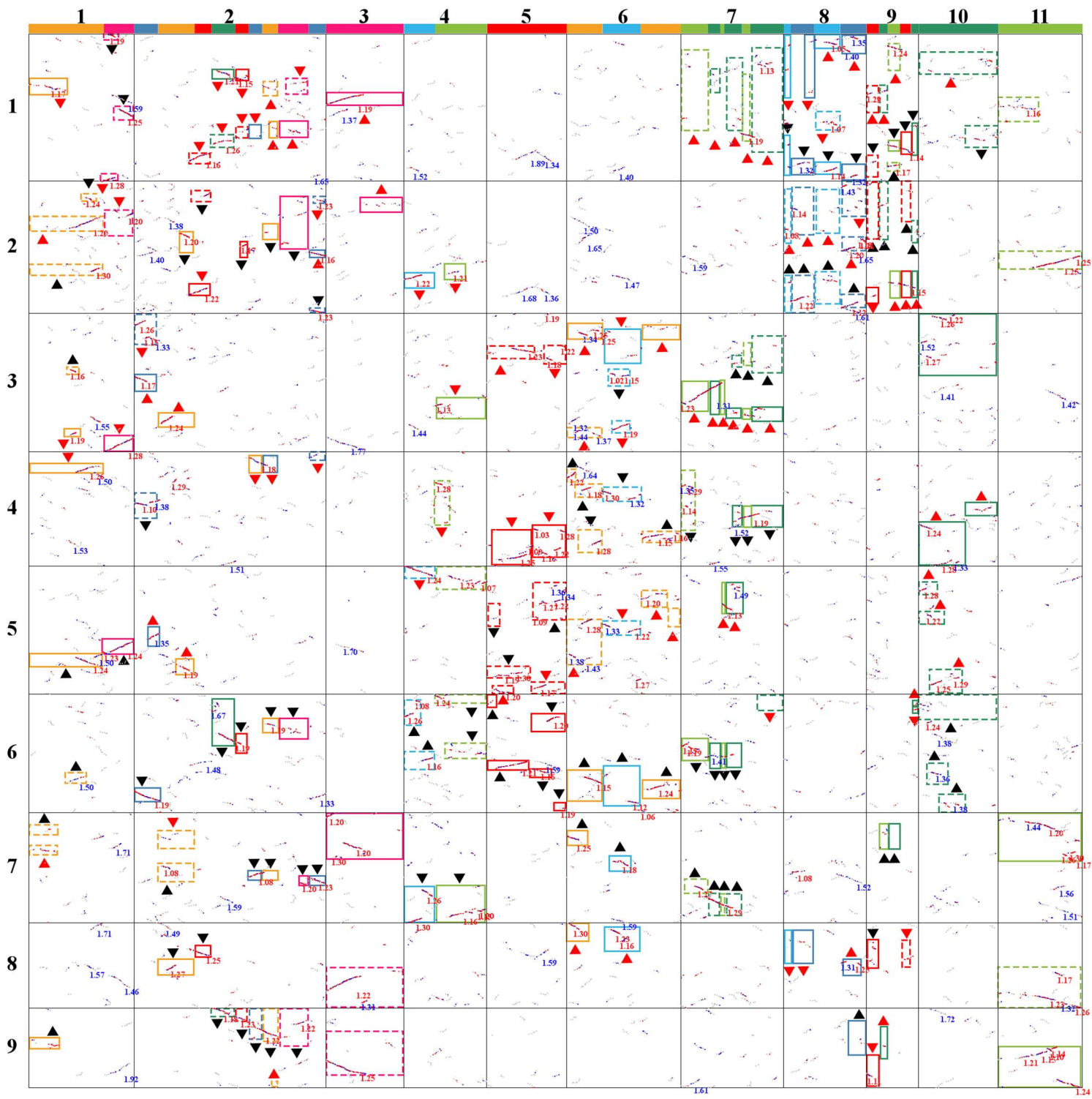

# Coffee.chr<sub>3</sub>

0-10.49 Mbp

## Carrot.chr

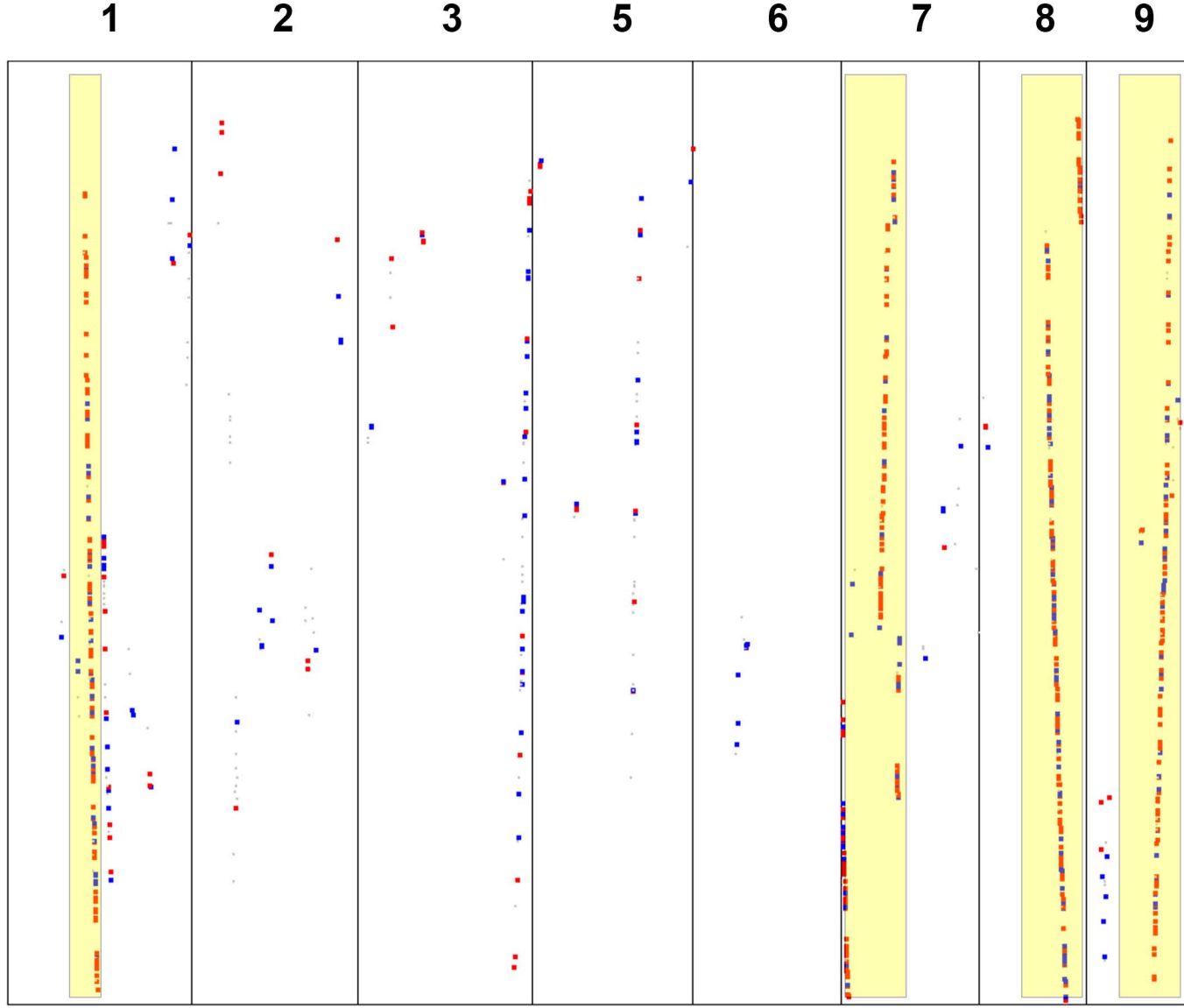

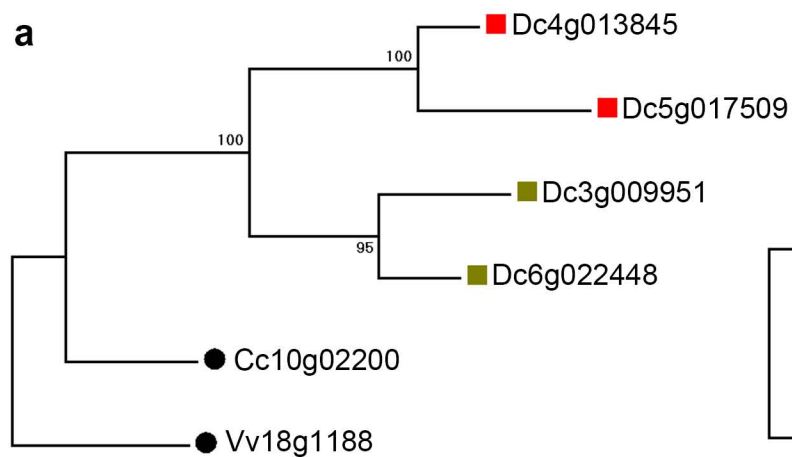

0.1

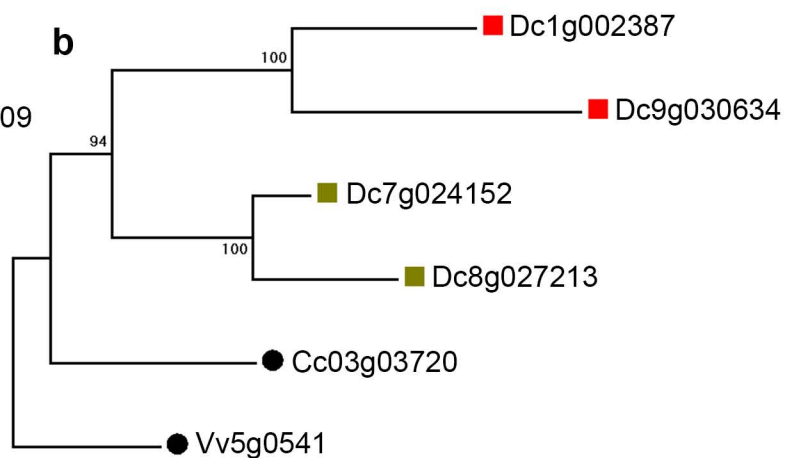

0.1

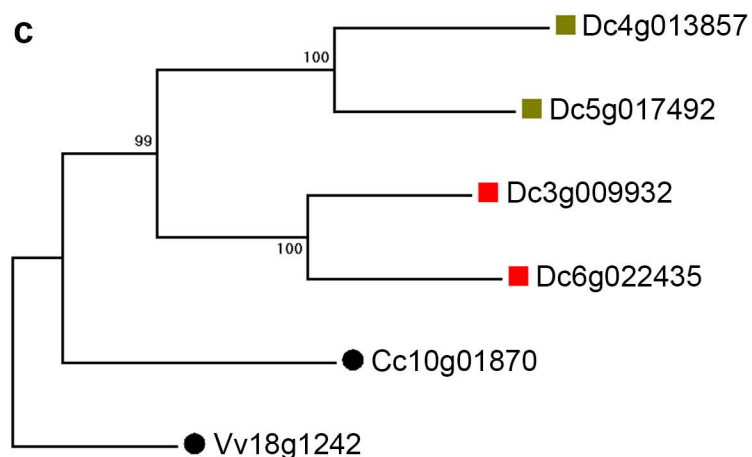

0.05

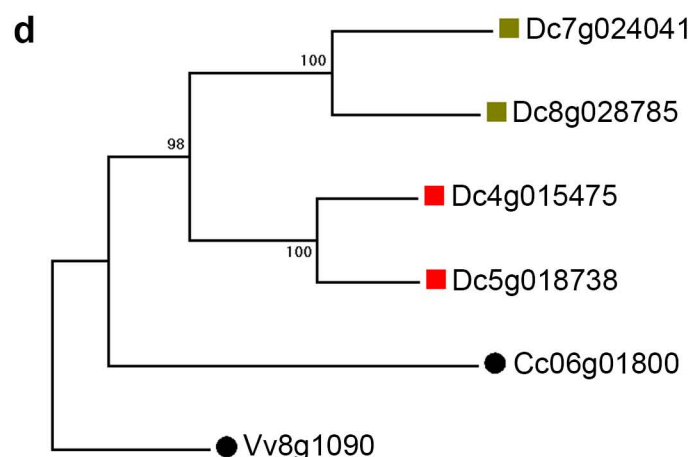

0.1

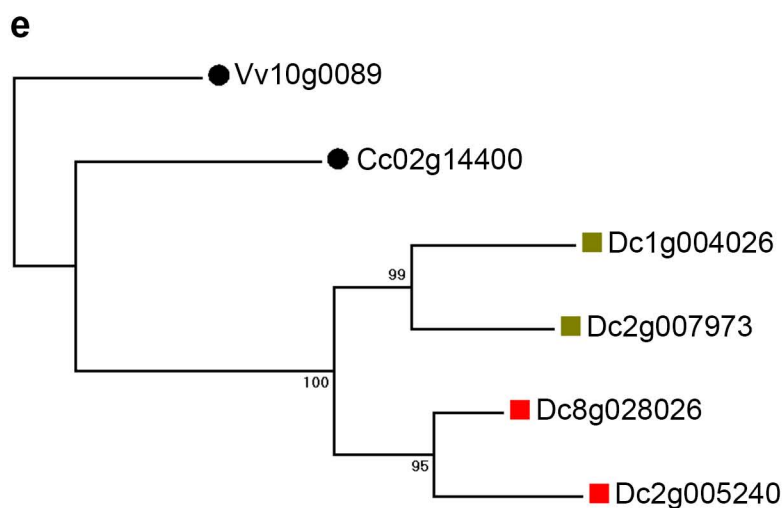

0.1

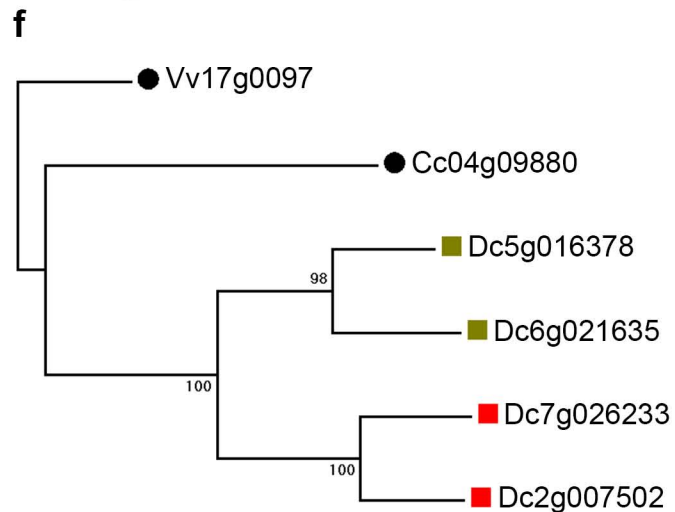

0.1

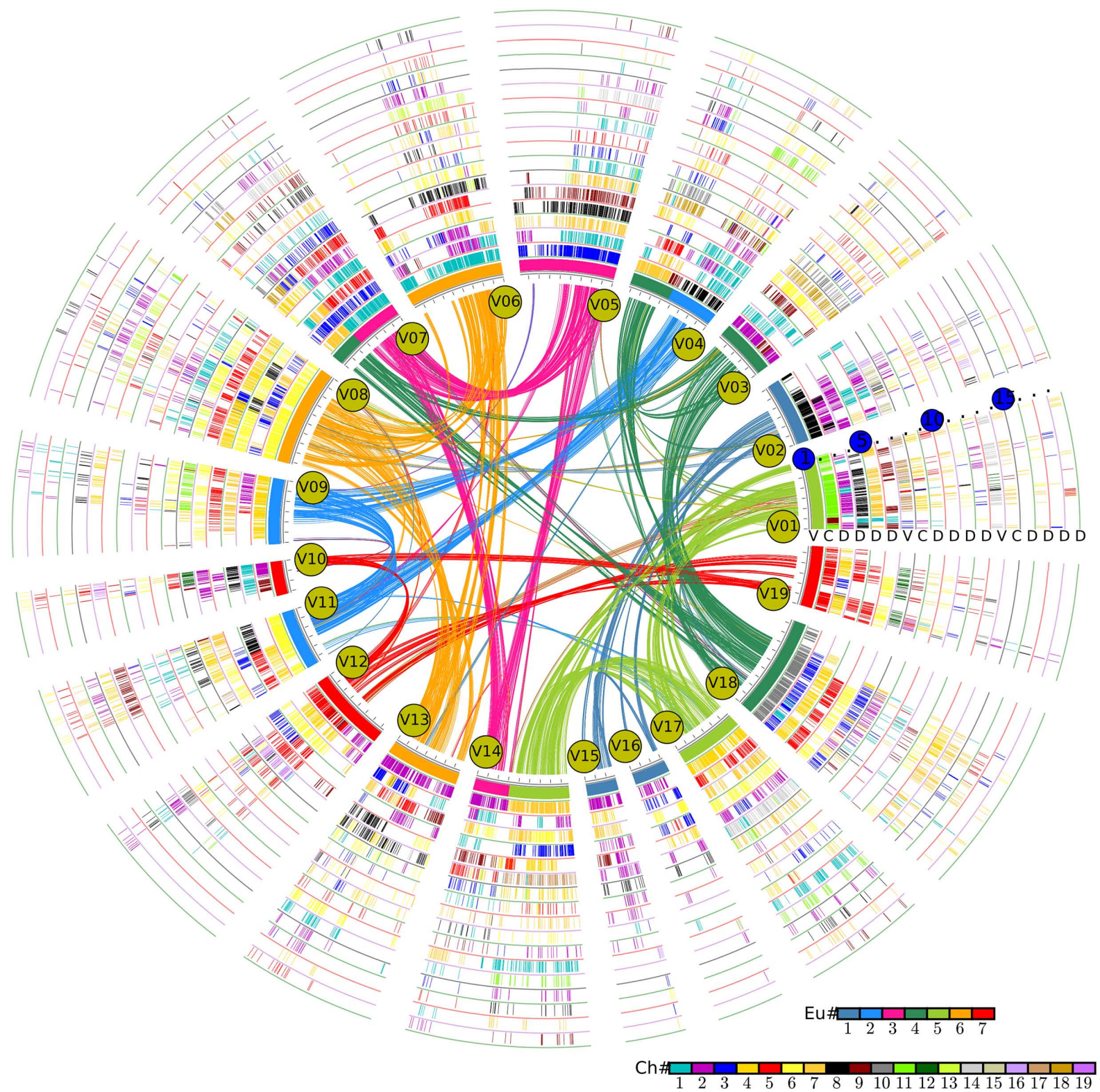

# Lettuce

1

2

3

4

5

6

7

8

9

1

2

3

4

5

6

7

8

9

10

11

12

13

14

15

16

17

18

19

Grape

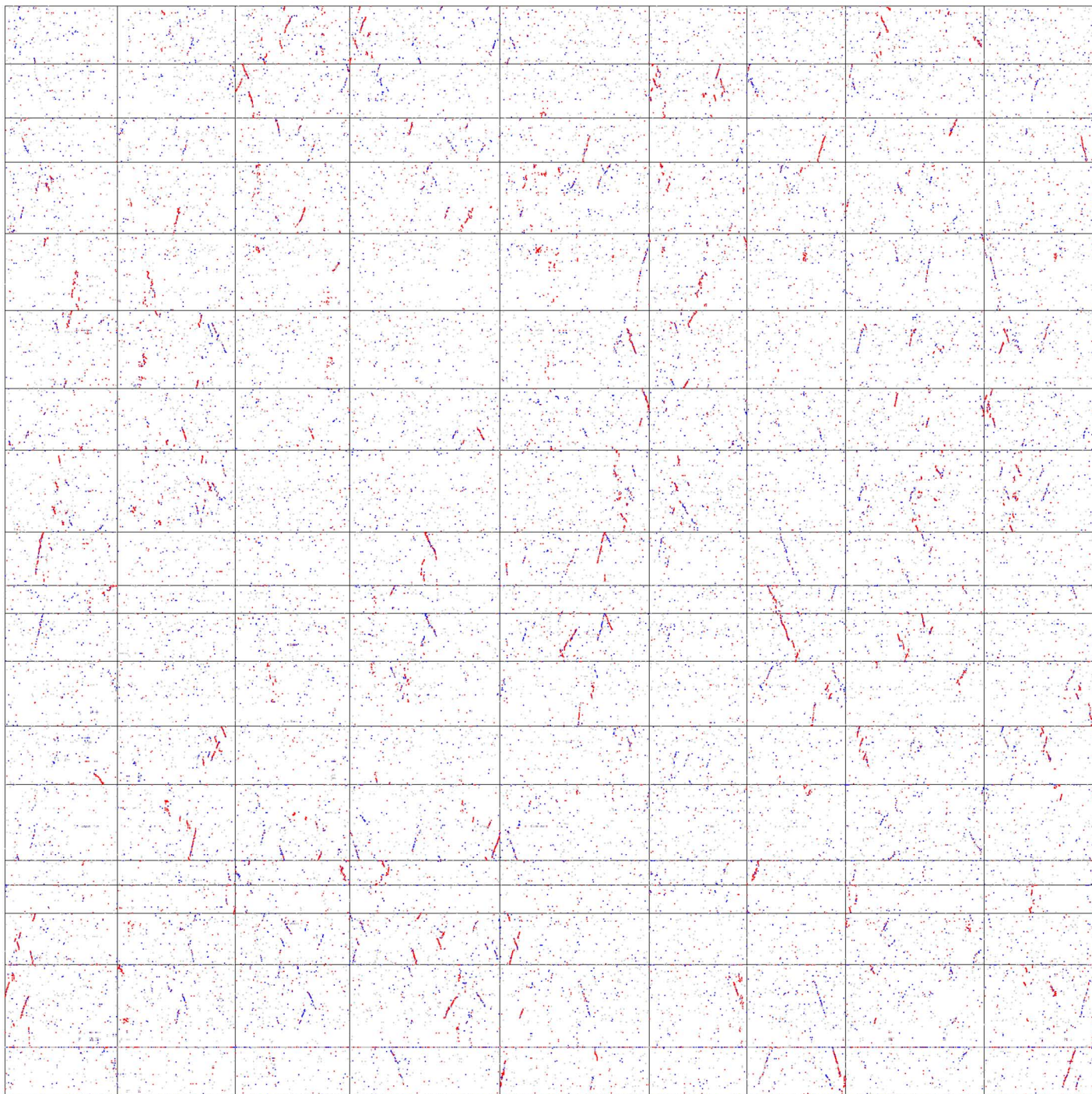

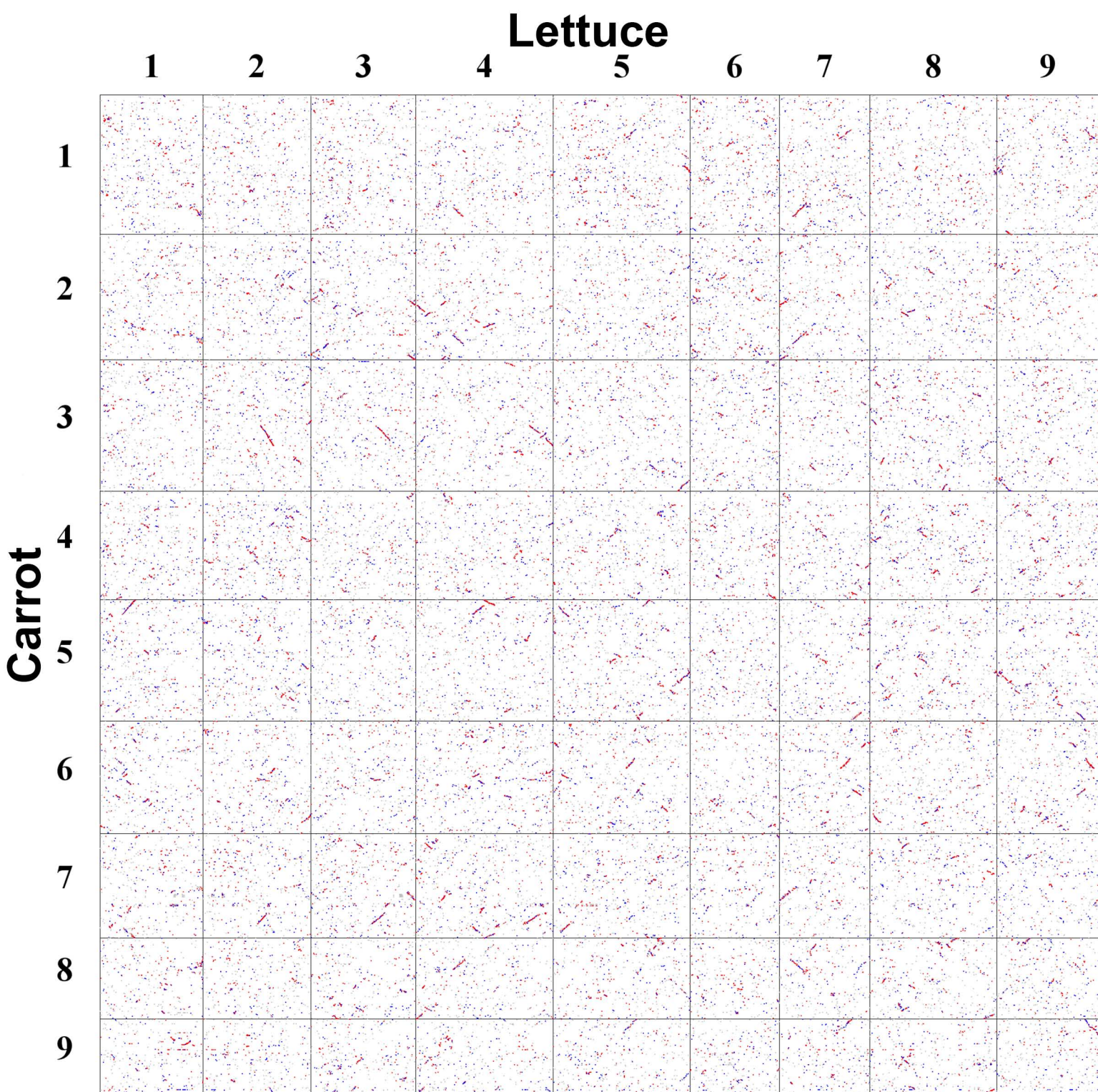

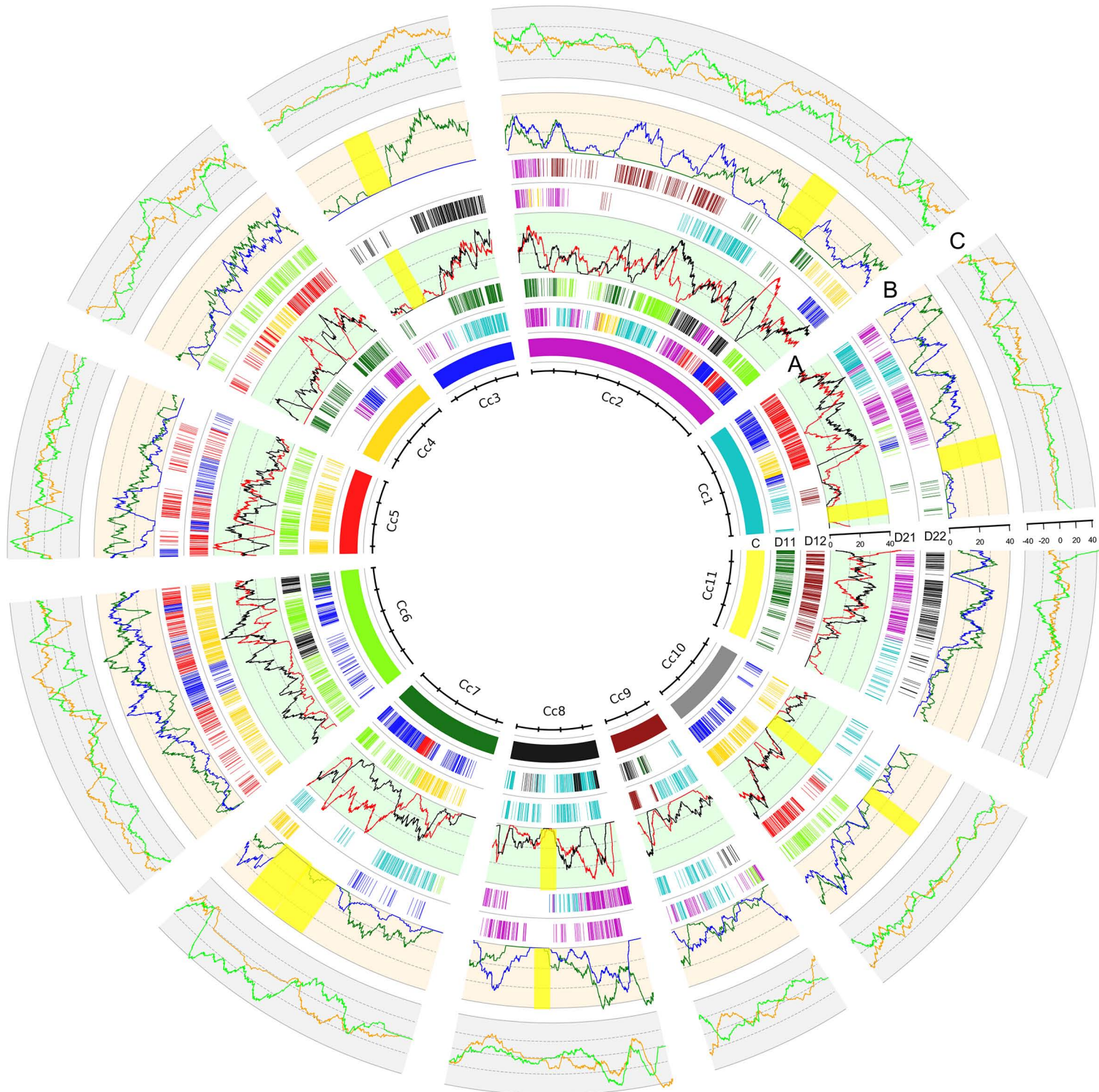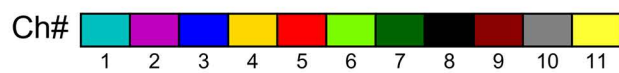

**a**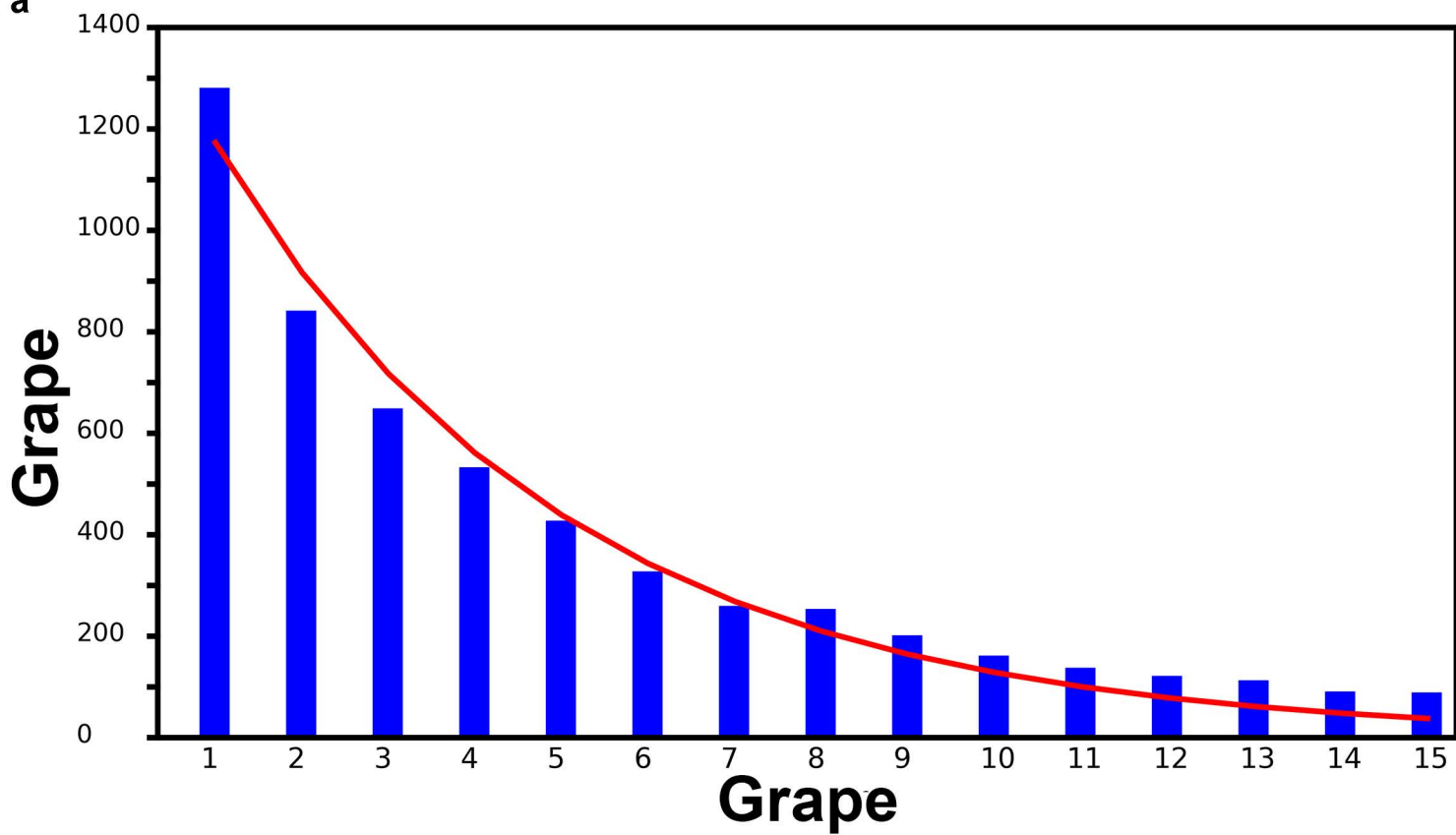**b**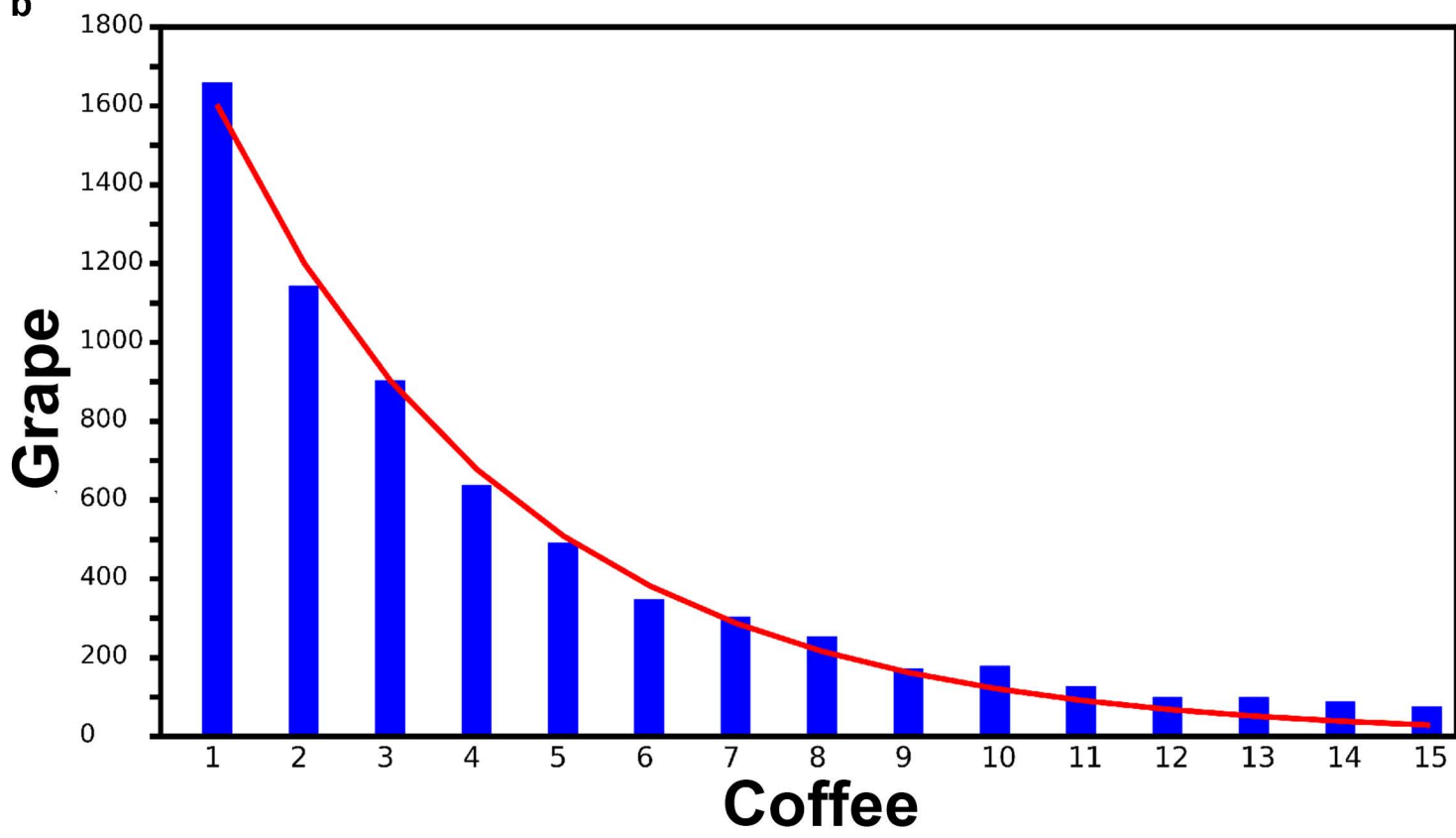

Supplement: Supplementary file 1 — Additional file 1: Figure S1. Homologous dotplot within carrot genome. Figure S2. Homologous dotplot between grape and carrot genomes. Figure S3. Homologous dotplot between coffee and carrot genomes. Figure S4. Examples of homologous gene dotplots between carrot and coffee. Figure S5. Trees with topology supporting the Dc-α and Dc-β.a-f. Figure S6. Alignment of carrot, coffee and grape genomes. Figure S7. Homologous dotplot between grape and lettuce. Figure S8. Homologous dotplot between carrot and lettuce. Figure S9. Homologous alignments and carrot subgenome gene retention along corresponding orthologous coffee chromosomes. Details in Fig. 4. Figure S10. Fitting a geometric distribution and gene loss rates in carrot as to the grape and coffee. [file 12870_2020_2235_MOESM1_ESM.pdf]
