# Supplementary material for: Sequential Paleotetraploidization shaped the carrot genome
Source: BMC Plant Biol. 2020 Jan 31;20:52. doi: 10.1186/s12870-020-2235-7 (PMC6995200; doi:10.1186/s12870-020-2235-7)
Supplement: Supplementary file 2 — Additional file 2: Table S1. Number of homologous blocks and gene pairs within a genome or between genomes. Table S2. Number of homologous genes within a genome or between genomes. Table S3. Orthologous genomic regions between grape and carrot. Table S4. Orthologous genomic regions between coffee and carrot. Table S5 S6. S7. Paralogous, orthologous and out-paralogous gene pairs within a genome or between genomes. Table S10. Kernel function analysis of Ks distribution related to duplication events within each genome and between genomes (before evolutionary rate correction.) Table S11. Kernel function analysis of Ks distribution related to duplication events within each genome and between genomes (after evolutionary rate correction). Table S12. Homologous depth within carrot, coffee and grape genome. Table S13. Intergenomic homologous depth of carrotgenome with grape or coffee as reference. Table S14. Intergenomic homologous depth of carrotgenome with grape or coffee as reference. Table S15. Carrot gene loss rates and gene translocation with coffee as reference genome. Table S16. The observed distribution of gene loss and translocation numbers fitted by using different density curves of geometry distribution. Table S19. Carotenoid accumulation gene family. Table S20. Information of genomic data. [file 12870_2020_2235_MOESM2_ESM.pdf]

Sequential paleotetraploidization shaped carrot genome

Additional tables

Table S1. Number of homologous blocks and gene pairs within a genome or between genomes.

Homologous Blocks within and among genome	BL[a] > 4	BL > 10	BL > 20	BL > 50	ACGP[b]	LDB[c]	LDB on chromosomes
Vitis vinifera	1,831/169	1,230/55	783/24	111/2	10.83, 22.36, 32.63, 55.50	61	VV05-VV07
Coffea canephora	2,436/270	1,301/45	1,048/26	408/6	9.02, 28.91, 40.31, 68.00	95	CC01-CC06
Daucus carota	7,142/745	3,913/166	2,545/62	1,192/15	9.59, 23.57, 41.05, 79.47	122	DC01-DC02
V. vinifera* vs *C. canephora	15,712/1,283	10,288/232	9,108/145	6,325/56	12.25, 44.34, 62.81, 112.95	326	VV05-CC03
V. vinifera* vs *D. carota	17,427/1,684	10,503/442	7,032/181	2,548/34	10.35, 23.76, 38.85, 74.94	142	VV05-DC08
C. canephora* vs *D. carota	20,939/2,001	12,346/423	9,378/200	5,352/64	10.46, 29.19, 46.89, 83.63	194	CC03-DC08

[a]BL: block_length; [b]ACGP: average colinear gene pairs respectively per block; [c]LDB: number of colinear gene pairs reside in longest duplicated block

Table S2. Number of homologous genes within a genome or between genomes.

Homologous Blocks within and among genome	BL[a]> 4	BL> 10	BL> 20	BL> 50	LDB[b]	LDB on chromosomes
Vitis vinifera	3,049	2,184	1,469	222	61	VV05-VV07
Coffea canephora	3,701	2231	1812	800	95	CC01-CC06
Daucus carota	8904	5957	4232	1967	122	DC02-DC02
V. vinifera* vs *C. canephora	9887 vs 9994	8364 vs 8309	7634 vs 7632	5836 vs 5814	326	VV05-CC03
V. vinifera* vs *D. carota	9270 vs 12133	7048 vs 9031	5294 vs 6681	2120 vs 2548	142	VV05- DC08
C. canephora* vs *D. carota	10551 vs 13819	7998 vs 10622	6645 vs 8675	4359 vs 5345	194	CC03-DC08

[a]BL: block_length; [b]LDB: number of colinear gene pairs reside in longest duplicated block

Table S3. Orthologous genomic regions between grape and carrot.

Grape chromosomes	Corresponding orthologous regions between grape and carrot			
**Vv1**	A1:Vv01:0-1,327:Dc01:1,726-2,318<br>A1:Vv01:0-1,327:Dc02:2,122-2,761	A2:Vv01:0-1327:Dc01:3091-3528<br>A2:Vv01:0-1327:Dc08:0-1207	A3:Vv01:0-1327:Dc07:0-1466<br>A3:Vv01:0-211:Dc01:644-979	A4:Vv01:0-211:Dc01:3257-4301<br>A4:Vv01:0-400:Dc09:993-1323<br>A4:Vv01:380-1327:Dc09:993-1552<br>A4:Vv01:674-1327:Dc09:1934-2316
**Vv2**	A1:Vv02:0-741:Dc02:1,432-2,096<br>A1:Vv02:0-443:Dc02:1,994-2,200<br>A1:Vv02:0-1,237:Dc08:1,207-2,157	A2:Vv02:0-1237:Dc02:3272-3758<br>A2:Vv02:0-433:Dc02:3630-3860	A3:Vv02:0-244:Dc07:1801-2161<br>A3:Vv02:247-1237:Dc01:0-567	A4:Vv02:0-1237:Dc01:3657-4301<br>A4:Vv02:0-1000:Dc02:0-409<br>A4:Vv02:392-887:Dc02:716-971
**Vv3**	A1:Vv03:371-1,000:Dc01:901-1,442<br>A1:Vv01:0-438:Dc06:2,810-3,066	A2:Vv03:0-1000:Dc01:2601-3168	A3:Vv03:0-1000:Dc09:0-1000	A4:Vv03:0-542:Dc06:3015-3270<br>A4:Vv03:417-1000:Dc06:1865-2402
**Vv4**	A1:Vv04:0-995:Dc01:0-1,159<br>A1:Vv04:1,348-1638:Dc01:0-1,159<br>A1:Vv04:518-1,140:Dc07:0-900<br>A1:Vv04:1,016-1,638:Dc07:2,264-3216	A2:Vv04:0-954:Dc01:3760-4198<br>A2:Vv04:394-995:Dc02:562-1559<br>A2:Vv04:829-1638:Dc05:0-1405	A3:Vv04:0-871:Dc01:2292-2730<br>A3:Vv04:0-500:Dc01:3065-3322<br>A3:Vv04:800-1638:Dc03:0-256<br>A3:Vv04:601-1638:Dc03:2148-3119	A4:Vv04:0-1600:Dc02:2812-3579<br>A4:Vv04:800-1683:Dc06:1431-1967<br>A4:Vv04:1000-1683:Dc04:1438-1824
**Vv5**	A1:Vv05:0-1,131:Dc01:1,623-2,138<br>A1:Vv05:1,131-1,748:Dc02:0-920	A2:Vv05:0-1748:Dc07:0-1544	A3:Vv05:0-1748:Dc08:0-1335	A4:Vv05:0-1700:Dc09:1349-2316<br>A4:Vv05:800-1748:Dc09:0-1171<br>A4:Vv05:900-1748:Dc08:1644-2491
**Vv6**	A1:Vv06:0-1,779:Dc01:1,288-1,751	A2:Vv06:0-290:Dc07:0-1600<br>A2:Vv06:0-1000:Dc02:0-818	A3:Vv06:0-1779:Dc04:2671-3110	A4:Vv06:0-848:Dc05:2631-2964<br>A4:Vv06:786-1779:Dc05:1942-3730

	A1:Vv06:265-1,779:Dc02:997-1,406<br>A1:Vv06:1,262-1,799:Dc09:0-1,800<br>A1:Vv07:0-1,309:Dc03:3,529-4,040	A2:Vv06:0-1779:Dc02:2428-2761<br>A2:Vv06:786-1779:Dc03:3426-4040<br>A2:Vv06:800-1779:Dc07:1600		
**Vv7**	A1:Vv07:1,140-1,409:Dc03:0-2,000<br>A1:Vv07:700-1,409:Dc03:1,943-2,327<br>A1:Vv07:1,181-1,409:Dc03:2,608-3,068	A2:Vv07:0-1409:Dc05:2044-2606<br>A2:Vv07:850-1409:Dc07:1600-3216<br>A2:Vv07:1098-1409:Dc01:489-1004	A3:Vv07:0-1036:Dc01:2060-2575<br>A3:Vv07:539-1077:Dc01:0-747<br>A3:Vv07:974-1409:Dc06:0-1865	A4:Vv07:0-912:Dc02:741-1329<br>A4:Vv07:684-1400:Dc01:3915-4301<br>A4:Vv07:808-1409:Dc04:0-800<br>A4:Vv07:800-1409:Dc04:1233-2000
**Vv8**	A1:Vv08:0-1,867:Dc03:143-918<br>A1:Vv08:0-1,867:Dc07:0-952	A2:Vv08:0-1867:Dc06:0-1780<br>A2:Vv08:0-1867:Dc08:2209-2491	A3:Vv08:0-1867:Dc04:488-1053<br>A3:Vv08:0-1867:Dc04:2055-2414<br>A3:Vv08:0-1867:Dc04:2517-2877<br>A3:Vv08:851-1390:Dc03:2632-4040	A4:Vv08:0-643:Dc05:2325-2759<br>A4:Vv08:975-1245:Dc05:2453-2785<br>A4:Vv08:0-1867:Dc05:0-1788
**Vv9**	A1:Vv09:0-1,221:Dc02:2,556-3,042	A1:Vv09:200-1221:Dc05:1533-1993<br>A2:Vv09:0-1221:Dc07:2018-3216	A3:Vv09:0-1221:Dc05:0-684	A4:Vv09:0-1221:Dc06:1405-3449
**Vv10**	A1:Vv10:0-632:Dc01:3,194-4,301	A2:Vv10:0-632:Dc08:1258-2400	A3:Vv10:0-1221:Dc02:0-1023<br>A3:Vv10:0-200:Dc04:0-500	A4:Vv10:0-1221:Dc02:2812-3860
**Vv11**	A1:Vv11:0-554:Dc03:2,448-2,836<br>A1:Vv11:383-1,107:Dc04:1,747-2,440	A2:Vv11:0-660:Dc08:1721-2491<br>A2:Vv11:383-1107:Dc05:1584-2146	A3:Vv11:0-1107:Dc03:306-1714	A4:Vv11:0-1000:Dc07:1132-1672<br>A4:Vv11:490-1107:Dc06:0-1686
**Vv12**	A1:Vv12:0-967:Dc04:0-568<br>A1:Vv12:658-1,481:Dc04:568-1,721<br>A1:Vv12:1,172-1,481:Dc02:1,713-2,200	A2:Vv12:0-1481:Dc06:1175-2223<br>A2:Vv12:1131-1481:Dc07:1904-2238	A3:Vv12:0-638:Dc03:300-1278<br>A3:Vv12:432-1481:Dc05:3449-3730<br>A3:Vv12:900-1481:Dc01:1159-1800	A4:Vv12:0-987:Dc05:485-3347<br>A4:Vv12:500-1481:Dc09:0-1000
**Vv13**	A1:Vv13:0-665:Dc03:2,914-3,34	A2:Vv13:0-644:Dc07:1389-1724	A3:Vv13:0-636:Dc03:1602-1977	A4:Vv13:0-727:Dc08:1002-2311

	1<br>A1:Vv13:166-768:Dc04:2,337-2,671<br>A1:Vv13:810-1,329:Dc01:2,498-2,962	A2:Vv13:166-997:Dc05:2836-3193<br>A2:Vv13:976-1329:Dc09:100-2316	A3:Vv13:540-1329:Dc02:1380-1789	A4:Vv13:976-1329:Dc06:2376-3000
**Vv14**	A1:Vv14:0-1,729:Dc01:2,395-3,000<br>A1:Vv14:0-1,729:Dc02:0-869<br>A1:Vv14:1,042-1,729:Dc02:1,559-2,122<br>A1:Vv14:750-1,062:Dc02:2,531-2,889	A2:Vv14:0-1500:Dc06:1788-2555<br>A2:Vv14:0-1729:Dc07:1878-2316<br>A2:Vv14:0-1062:Dc07:2316-3216	A3:Vv14:0-687:Dc01:0-1803<br>A3:Vv14:0-1729:258-700<br>A3:Vv14:750-1729:Dc03:2097-2710	A4:Vv14:479-1729:Dc04:1310-2414<br>A4:Vv14:667-937:Dc05:894-1431<br>A4:Vv14:0-1000:Dc09:0-916
**Vv15**	A1:Vv15:0-561:Dc04:2,954-3,339	A2:Vv15:0-561:Dc07:1647-3216	A3:Vv15:0-500:Dc02:0-946<br>A3:Vv15:0-561:Dc02:1994-2275	A4:Vv15:0-561:Dc02:3579-3860<br>A4:Vv15:0-500:Dc09:0-930
**Vv16**	A1:Vv16:0-647:Dc03:0-895	A2:Vv16:0-647:Dc06:0-1500	A3:Vv16:0-647:Dc03:1688-2199<br>A3:Vv16:0-647:Dc05:1814-2400	A4:Vv16:0-647:DC04:1464-2312
**Vv17**	A1:Vv17:0-542:Dc04:1,824-3,339<br>A1:Vv17:0-1,168:Dc05:0-792	A2:Vv17:0-1168:Dc07:2521-3216	A3:Vv17:0-1168:Dc06:1507-1891<br>A3:Vv17:0-1168:Dc06:3142-3449	A4:Vv17:0-292:Dc02:2531-2837<br>A4:Vv17:521-1168:Dc02:2122-2786<br>A4:Vv17:0-584:Dc03:2531-2838
**Vv18**	A1:Vv18:352-1,161:Dc03:0-256<br>A1:Vv18:0-725:Dc03:716-1,304<br>A1:Vv18:1,036-1,886:Dc03:665-1,764<br>A1:Vv18:20-725:Dc01:2,859-3,271	A2:Vv18:0-435:Dc04:1053-1824<br>A2:Vv18:100-1886:Dc04:0-514<br>A2:Vv18:953-1886:Dc04:0-1515	A3:Vv18:0-415:Dc05:0-3730<br>A3:Vv18:953-1886:Dc05:1226-1712<br>A3:Vv18:415-1244:Dc05:3117-3526	A4:Vv18:0-622:Dc06:3015-3449<br>A4:Vv18:0-2000:Dc06:0-434<br>A4:Vv18:0-1886:Dc06:996-1303<br>A4:Vv18:100-1886:Dc06:2376-2759<br>A4:Vv18:1205-1886:Dc06:2970-3449
**Vv19**	A1:Vv19:248-1,135:Dc03:1,202-1,534<br>A1:Vv19:0-1,135:Dc04:283-1,233	A2:Vv19:0-1135:Dc05:0-1661	A3:Vv19:0-1135:Dc05:3321-3730	A4:Vv19:0-1135:Dc06:869-1329<br>A4:Vv19:206-1135:Dc06:2453-3449

Each grape genomic region corresponds to four or fewer independent carrot regions, denoted from A1-A4. The chromosome identification

numbers and ranges (measured in genes) that contain orthologous genes from both species have been shown.

Table S4. Orthologous genomic regions between coffee and carrot.

**Coffee chromosomes**	**Corresponding orthologous regions between coffee and carrot**			
**Cc1**	A1:Cc01:0-1100:Dc01:0-2150<br>A1:Cc01:0-1100:Dc03:2150-4301<br>A1:Cc01:1514-2198:Dc03:3457-4040<br>A1:Cc01:500-2198:Dc04:2657-3339	A2:Cc01:0-1000:Dc09:886-2316<br>A2:Cc01:855-2198:Dc05:2019-3303	A3:Cc01:1000-2200:Dc01:0-542<br>A3:Cc01:1661-2198:Dc01:2032-2574<br>A3:Cc01:0-1470:Dc02:0-655<br>A3:Cc01:800-2000:Dc02:1900-3860<br>A3:Cc01:0-2198:Dc03:1000-3000<br>A3:Cc01:800-2000:Dc06:1700-3449<br>A3:Cc01:0-1100:Dc07:0-821	A4:Cc01:0-1100:Dc07:822-1700<br>A4:Cc01:0-1472:Dc02:650-1482<br>A4:Cc01:1500-2198:Dc02:620-1482<br>A4:Cc01:1100-2198:Dc01:3960-4301
**Cc2**	A1:Cc02:0-878:Dc03:1335-3355<br>A1:Cc02:0-634:Dc05:1848-2327<br>A1:Cc02:805-1220:Dc05:2738-3730<br>A1:Cc02:1098-1659:Dc02:2972-3518<br>A1:Cc02:1660-2439:Dc01:785-1673<br>A1:Cc02:2268-3049:Dc04:2828-3339<br>A1:Cc02:2634-3610:Dc01:2594-4000<br>A1:Cc02:2610-4000:Dc02:1298-3000	A2:Cc02:0-561:Dc06:0-1500<br>A2:Cc02:561-1683:Dc08:0-2116<br>A2:Cc02:854-1224:Dc02:0-1879<br>A2:Cc02:1512-4000:Dc06:1878-3449<br>A2:Cc02:2098-4000:Dc07:1608-2326	A3:Cc02:0-310:Dc03:0-993<br>A3:Cc02:2941-4000:Dc01:1000-1820<br>A3:Cc02:0-2000:Dc07:0-992<br>A3:Cc02:400-2585:Dc01:2970-4301<br>A3:Cc02:2000-4000:Dc09:2009-2316<br>A3:Cc02:2902-4000:Dc02:0-1127<br>A3:Cc02:3390-4000:Dc04:2800-3339	A4:Cc02:1235-1600:Dc02:0-500<br>A4:Cc02:0-610:Dc04:0-2249<br>A4:Cc02:0-1573:Dc07:1266-1779<br>A4:Cc02:200-4000:Dc09:0-1090<br>A4:Cc02:3585-4000:Dc02:3655-3860
**Cc3**	A1:Cc03:0-1632:Dc01:1000-2185	A2:Cc03:0-1632:Dc07:0-2000	A3:Cc03:0-1632:Dc08:0-2000	A4:Cc03:0-1632:Dc09:732-2316

	A1:Cc03:500-1632:Dc02:0-1500			
**Cc4**	A1:Cc04:0-1241:Dc02:2528-3108<br>A1:Cc04:438-1727:Dc03:0-4040	A2:Cc04:0-1727:Dc07:2121-3216	A3:Cc04:486-1727:Dc04:0-3339<br>A3:Cc04:0-1727:Dc05:0-700	A4:Cc04:0-1727:Dc06:1298-3449
**Cc5**	A1:Cc05:0-1661:Dc04:0-1295	A2:Cc05:879-1295:Dc06:0-1400<br>A2:Cc05:0-1300:Dc06:1195-3449<br>A2:Cc05:1000-1661:Dc06:1700-3449<br>A2:Cc05:1000-1661:Dc06:0-400	A3:Cc05:0-635:Dc05:3422-3730<br>A3:Cc05:0-1075:Dc03:0-1438<br>A3:Cc05:1172-1466:Dc03:1130-1500<br>A3:Cc05:831-1661:Dc05:0-2000	A4:Cc05:1466-1661:Dc03:0-1432<br>A4:Cc05:0-300:Dc05:811-1000<br>A4:Cc05:0-879:Dc05:2738-3354<br>A4:Cc05:879-1661:Dc05:3319-3730
**Cc6**	A1:Cc06:0-2389:Dc03:0-1438<br>A1:Cc06:0-800:Dc07:0-1026<br>A1:Cc06:500-2000:Dc07:0-1847	A2:Cc06:0-1536:Dc08:1672-2491<br>A2:Cc06:0-1073:Dc06:0-1725<br>A2:Cc06:1000-2389:Dc06:0-1059	A3:Cc06:0-281:Dc04:0-3339<br>A3:Cc06:0-2389:Dc04:1772-2385<br>A3:Cc06:0-2389:Dc04:545-1056<br>A3:Cc06:756-2389:Dc03:1541-2191	A4:Cc06:0-2389:Dc03:3150-3800<br>A4:Cc06:0-2389:Dc05:616-2156<br>A4:Cc06:0-268:Dc05:2224-2806
**Cc7**	A1:Cc07:0-2146:Dc03:1883-4000<br>A1:Cc07:0-2146:Dc05:958-1403	A2:Cc07:1024-2176:Dc04:1193-3000<br>A2:Cc07:0-561:Dc06:1742-2117<br>A2:Cc07:439-1195:Dc06:1503-2110	A3:Cc07:0-2146:Dc01:0-3140<br>A3:Cc07:1700-2146:Dc06:0-300	A4:Cc07:0-366:Dc04:0-3339<br>A4:Cc07:1658-2146:Dc03:0-1154<br>A4:Cc07:854-2146:Dc03:1155-1746<br>A4:Cc07:0-2000:Dc07:1600-2804
**Cc8**	A1:Cc08:0-1718:Dc01:0-1946<br>A1:Cc08:196-1718:Dc08:1092-2116	A2:Cc08:0-1718:Dc01:3038-4301<br>A2:Cc08:1497-1718	A3:Cc08:491-1227:Dc01:2321-2901<br>A3:Cc08:0-1718:Dc02:0-2085	A4:Cc08:0-1718:Dc02:2664-3758
**Cc9**	A1:Cc09:0-1094:Dc07:513-855<br>A1:Cc09:0-535:Dc08:0-2491<br>A1:Cc09:0-1094:Dc02:2000-3860	A2:Cc09:0-535:Dc09:0-2316<br>A2:Cc09:365-1094:Dc01:2357-3860	A3:Cc09:0-875:Dc01:614-2219<br>A3:Cc09:729-1094:Dc08:0-1638	A4:Cc09:0-802:Dc01:3516-4028<br>A4:Cc09:0-1094:Dc02:0-1810<br>A4:Cc09:500-1094:Dc06:2834-3449
**Cc10**	A1:Cc10:0-1653:Dc03:0-1849	A2:Cc10:0-899:Dc04:0-1295<br>A2:Cc10:851-1653:Dc04:1158-3339	A3:Cc10:0-267:Dc05:0-1700<br>A3:Cc10:0-500:Dc05:1300-2000<br>A3:Cc10:0-948:Dc05:2943-3730<br>A3:Cc10:800-1653:Dc01:0-1092	A4:Cc10:0-340:Dc06:2493-3449<br>A4:Cc10:0-1094:Dc06:0-1366<br>A4:Cc10:1021-1653:Dc01:2765-4000
**Cc11**	A1:Cc11:0-1753:Dc07:0-1847	A2:Cc11:0-1753:Dc09:0-2316	A3:Cc11:0-1000:Dc01:1604-2697<br>A3:Cc11:0-1753:Dc02:2050-2664	A4:Cc11:0-1753:Dc08:0-1491

Each coffee genomic region corresponds to four or fewer independent carrot regions, denoted from A1-A4. The chromosome identification

numbers and ranges (measured in genes) that contain orthologous genes from both species have been shown.

Table S5. Paralogous, orthologous and out-paralogous gene pairs within a genome or between genomes.

Genomes	Grape	Coffee	Carrot
Grape	2424	7471	9639
Coffee	3759	1640	11256
Carrot	5713	7850	5511

Numbers on the main diagonal denote paralogous gene pairs within a genome, numbers above the diagonal denote orthologous gene pairs between two genomes, and numbers below the diagonal denote out-paralogous gene pairs between two genomes.

Table S6. Paralogous, orthologous and out-paralogous genes within a genome or between genomes.

Genomes	Grape	Coffee	Carrot
Grape	3853	7355/7387	7013/9096
Coffee	3023/3199	2768	8152/10907
Carrot	4324/3544	5480/4724	6777

See the legends of Supplementary Table S5. In non-diagonal cells, gene numbers in two corresponding species were shown, from vertical and horizontal lists respectively.

Table S7. Paralogous, orthologous and out-paralogous blocks within a genome or between genomes.

Genomes	Grape	Coffee	Carrot
Grape	86	152	537
Coffee	154	92	488
Carrot	396	467	224

See the legends of Supplementary Table S5. In non-diagonal cells, gene numbers in two corresponding species were shown, from vertical and horizontal lists respectively.

Table S10. Kernel function analysis of Ks distribution related to duplication events within each genome and between genomes (before

evolutionary rate correction).

Intragenomic/Intergenomic colinear gene pairs	Weight coefficient related to duplication event or speciation	Peak of Ks distribution (μ)	Deviation (σ)	Correction coefficient (λ)
**Grape ECH-related**	0.569	1.053	0.120	1
**Coffee ECH-related**	0.137	1.400	0.070	0.752
**Carrot ECH-related**	0.116	1.390	0.099	0.758
**Carrot Dc-β-related**	0.498	0.944	0.176	0.758
**Carrot Dc-α-related**	0.171	0.551	0.060	0.758
**Lettuce- ECH-related**	0.298	1.486	0.060	0.732
**Lettuce- sWGT-related**	0.322	0.872	0.131	0.732
**Grape-Coffee**	0.264	1.085	0.094	0.858
**Coffee-Carrot (Split)**	0.202	1.195	0.116	0.755
**Coffee-Carrot (ECH)**	0.139	1.496	0.129	0.755
**Lettuce-Carrot**	0.716	1.175	0.256	0.758

Table S11. Kernel function analysis of Ks distribution related to duplication events within each genome and between genomes (after evolutionary rate correction).

Intragenomic/Intergenomic colinear gene pairs	Weight coefficient related to duplication event or speciation	Peak of Ks distribution (μ)	Deviation (σ)	Correction coefficient (λ)
**Grape ECH-related**	0.569	1.053	0.120	1
**Coffee ECH-related**	0.224	1.053	0.070	0.752
**Carrot ECH-related**	0.058	1.053	0.049	0.758
**Carrot Dc-β-related**	0.631	0.703	0.148	0.758
**Carrot Dc-α-related**	0.139	0.421	0.049	0.758
**Lettuce- ECH-related**	0.331	1.053	1.131	0.732
**Lettuce- sWGT-related**	0.405	0.619	0.072	0.732
**Grape-Coffee**	0.331	0.935	0.094	0.858
**Coffee-Carrot (Split)**	0.666	0.901	0.180	0.755
**Coffee-Carrot (ECH)**	0.430	1.053	0.189	0.755

**Lettuce-Carrot**	0.658	0.898	0.209	0.758

Supplementary Table S12. Homologous depth within carrot, coffee and grape genome.

**Genome**<br>**Intergenomic homologous depth level**	***V. vinifera***	***C. canephora***	***D. carota***
**0**	9728/0(0.411)	10064/0(0.458)	6047/0(0.197)
**1**	6280/1118(0.266)	5797/993(0.264)	7628/1900(0.248)
**2**	7310/2912(0.309)	5940/2166(0.270)	7708/3449(0.251)
**3**	322/180(0.014)	170/87(0.008)	6480/4081(0.211)
**4**	7/8(0.000)		2101/1619(0.068)
**5**			548/534(0.018)
**6**			205/249(0.007)
**7**			25/28(0.001)

In each cell, we show the genes number in the reference genome and the corresponding homologous regions piled up to certain depth in the compared plant genome, and the percentage of genes covered in the compared genome.

Supplementary Table S13. Intergenomic homologous depth of carrot genome with grape or coffee as reference.

Intragenomic homologous depth level	In *D. carota* as to *V. vinifera*	In *D. carota* as to *C. canephora*
0	3392/0(0.143)	3578/0(0.163)
1	2398/481(0.101)	2044/365(0.093)
2	3013/1169(0.127)	2270/991(0.103)
3	3614/2197(0.153)	2979/1835(0.136)
4	3727/3030(0.158)	3474/3082(0.158)
5	2812/2702(0.119)	2769/2955(0.126)
6	2338/2502(0.099)	2286/2805(0.104)
7	1502/2027(0.064)	1463/2012(0.067)
8	682/1016(0.029)	736/1134(0.034)
9	154/242(0.007)	297/556(0.014)
10	15/30(0.001)	63/127(0.003)
11		3/7(0.000)
12		9/24(0.000)

In each cell, we show the genes number in the reference genome and the corresponding homologous regions piled up to certain depth in the compared plant genome, and the percentage of genes covered in the compared genome.

Table S14. Carrot gene loss rates and gene translocation with grape as reference genome.

Grape		Carrot homoelogous subgenomes					
**Chr #**	**Gene #**	**AAT- 1**	**AAT-2**	**AAT 1-2 difference**	**ART-1**	**ART-2**	**ART 1-2 difference**
1	1327	0.79	0.82	0.03	0.8	0.86	0.06
2	1237	0.71	0.79	0.08	0.86	0.82	0.04
3	1000	0.9	0.86	0.04	0.91	0.84	0.07
4	1638	0.84	0.83	0.01	0.84	0.9	0.06
5	1748	0.87	0.85	0.02	0.86	0.88	0.02
6	1779	0.85	0.86	0.01	0.9	0.8	0.1
7	1409	0.83	0.81	0.02	0.82	0.87	0.05
8	1867	0.86	0.81	0.05	0.78	0.85	0.07
9	1221	0.86	0.88	0.02	0.9	0.9	0
10	632	0.85	0.83	0.02	0.83	0.86	0.03
11	1107	0.88	0.82	0.06	0.9	0.88	0.02
12	1481	0.84	0.85	0.01	0.87	0.88	0.01
13	1329	0.81	0.86	0.05	0.87	0.88	0.01
14	1729	0.88	0.83	0.05	0.86	0.85	0.01
15	561	0.92	0.88	0.04	0.91	0.86	0.05
16	647	0.86	0.89	0.03	0.89	0.92	0.03
17	1168	0.83	0.84	0.01	0.86	0.89	0.03
18	1886	0.84	0.84	0	0.79	0.86	0.07
19	1135	0.81	0.82	0.01	0.88	0.84	0.04
Total/Aver.	24901	0.84	0.84	0.03	0.86	0.87	0.04

Table S15. Carrot gene loss rates and gene translocation with coffee as reference genome.

Coffee		Carrot homoelogous subgenomes					
**Chr #**	**Gene #**	**T1- A**	**T1-B**	**T1 A-B difference**	**T2-A**	**T2-B**	**T2 A-B difference**
1	2198	0.85	0.74	0.11	0.86	0.64	0.22
2	4000	0.80	0.80	0	0.89	0.60	0.29
3	1632	0.85	0.81	0.04	0.85	0.64	0.21
4	1727	0.78	0.83	0.05	0.84	0.62	0.22
5	1661	0.82	0.84	0.02	0.87	0.62	0.25
6	2389	0.85	0.80	0.05	0.81	0.54	0.27
7	2146	0.80	0.85	0.05	0.77	0.54	0.23
8	1718	0.78	0.86	0.08	0.83	0.61	0.22
9	1094	0.86	0.85	0.01	0.85	0.72	0.13
10	1653	0.80	0.82	0.02	0.84	0.67	0.17
11	1753	0.87	0.81	0.06	0.87	0.64	0.23
Total/Aver.	21971	0.82	0.82	0.04	0.84	0.62	0.22

Table S16. The observed distribution of gene loss and translocation numbers fitted by using different density curves of geometry distribution.

Reference genome	Genomes	Parameter of geometry distribution	Fitness (R-square)	P-value (F-test)
Vitis Vinifera	*Daucus Carota*	0.2205	0.9797	0.9306
Coffea canephora	*Daucus Carota*	0.2489	0.9941	0.9259

Table S19. Carotenoid accumulation gene family.

Name	original number	Carrot				Grape		Coffee	
		Homologous (evalua<=1e-10,Score >=150)	related to γ-duplication	related to Dc-β duplication	related to Dc-α duplication	Homologous (evalua<=1e-10,Score >=150)	related to γ-duplication	Homologous (evalua<=1e-10,Score >=150)	related to γ-duplication
DXS	4	4	2(50.00%)	4(100.00%)	3(75.00%)	9	2(22.22%)	4	3(75.00%)
DXR	1	1	0(-)	1(100.00%)	1(100.00%)	1	1(100.00%)	2	2(100.00%)
MCT	2	2	0(-)	1(50.00%)	0(-)	1	0(-)	1	0(-)
CMK	1	1	0(-)	0(-)	0(-)	1	0(-)	1	0(-)
MTS	1	1	0(-)	1(100.00%)	1(100.00%)	1	1(100.00%)	1	1(100.00%)
HDS	2	2	1(50.00%)	2(100.00%)	1(50.00%)	1	1(100.00%)	1	1(100.00%)
HDR	3	3	0(-)	3(100.00%)	3(100.00%)	1	1(100.00%)	1	1(100.00%)
IPPI	2	2	0(-)	1(50.00%)	1(50.00%)	1	0(-)	1	1(100.00%)
GPPS	1	4	0(-)	3(75.00%)	3(75.00%)	2	0(-)	2	1(50.00%)
GGPS	6	7	2(28.57%)	6(85.71%)	5(71.43%)	5	4(80.00%)	3	3(100.00%)
PSY	3	3	1(33.33%)	2(66.67%)	1(33.33%)	3	1(33.33%)	2	1(50.00%)
CCD	6	17	0(-)	15(88.24%	14(82.35%	14	6(42.86%)	9	9(100.00%)
PDS	1	1	0(-)	1(100.00%)	1(100.00%)	1	1(100.00%)	1	1(100.00%)
ZISO	1	1	0(-)	1(100.00%)	1(100.00%)	1	0(-)	1	0(-)
CRTISO	2	2	0(-)	2(100.00%)	1(50.00%)	2	0(-)	2	2(100.00%)
ZDS	2	2	0(-)	1(50.00%)	1(50.00%)	2	2(100.00%)	2	1(50.00%)
LCYE	1	3	0(-)	3(100.00%)	2(66.67%)	3	1(33.33%)	3	3(100.00%)
LCYB	1	3	0(-)	3(100.00%)	2(66.67%)	3	1(33.33%)	3	3(100.00%)
CYP97B3	1	4	1(25.00%)	4(100.00%)	4(100.00%)	6	2(33.33%)	6	4(66.67%)
BCH1/C	1	3	0(-)	3(100.00%)	2(66.67%)	2	1(50.00%)	1	1(100.00%)

HXB									
BCH2	1	3	0(-)	3(100.00%)	2(66.67%)	2	1(50.00%)	1	1(100.00%)
CHXE	1	5	1(20.00%)	5(100.00%)	5(100.00%)	8	3(37.50%)	6	4(66.67%)
VDE	1	1	0(-)	1(100.00%)	1(100.00%)	1	0(-)	1	0(-)
ZEP	1	2	1(50.00%)	2(100.00%)	1(50.00%)	2	1(50.00%)	1	1(100.00%)
NCED	9	15	0(-)	14(93.33%)	13(86.67%)	11	4(36.36%)	6	6(100.00%)

Table S20. Information of genomic data.

Order	Species name	Common name	Version	Data source	Genes	Anchored Genes[a]	Reference
**1**	***Vitis vinifera L***	**Grape**	Genoscope.12X	JGI (https://phytozome.jgi.doe.gov/pz/portal.html#!info?alias=Org_Vvinifera)	37,829	24,901	(Jaillon *et al.*, 2007)
**2**	***Coffea canephora***	**Coffee**	v1.0	(http://coffee-genome.org/)	25574	21,971	(Denoeud *et al.*, 2014)
**3**	***Daucus carota L.***	**Carrot**	v2.0	JGI (https://phytozome.jgi.doe.gov/pz/portal.html#!info?alias=Org_Dcarota)	32,113	30,742	(Iorizzo *et al.*, 2016)

References

Denoeud, F., Carreteropaulet, L., Dereeper, A., Droc, G., Guyot, R., Pietrella, M., Zheng, C., Alberti, A., Anthony, F. and Aprea, G. (2014) The coffee genome provides insight into the convergent evolution of caffeine biosynthesis. *Science*, **345**, 1181.

Iorizzo, M., Ellison, S., Senalik, D., Zeng, P., Satapoomin, P., Huang, J., Bowman, M., Iovene, M., Sanseverino, W., Cavagnaro, P., Yildiz, M., Macko-Podgorni, A., Moranska, E., Grzebelus, E., Grzebelus, D., Ashrafi, H., Zheng, Z., Cheng, S., Spooner, D., Van Deynze, A. and Simon, P. (2016) A high-quality carrot genome assembly provides new insights into carotenoid accumulation and asterid genome evolution. *Nat Genet*, **48**, 657-666.

Jaillon, O., Aury, J.M., Noel, B., Policriti, A., Clepet, C., Casagrande, A., Choisne, N., Aubourg, S., Vitulo, N., Jubin, C., Vezzi, A., Legeai, F., Hugueney, P., Dasilva, C., Horner, D., Mica, E., Jublot, D., Poulain, J., Bruyere, C., Billault, A., Segurens, B., Gouyvenoux, M., Ugarte, E., Cattonaro, F., Anthouard, V., Vico, V., Del Fabbro, C., Alaux, M., Di Gaspero, G., Dumas, V., Felice, N., Paillard, S., Juman, I., Moroldo, M., Scalabrin, S., Canaguier, A., Le Clainche, I., Malacrida, G., Durand, E., Pesole, G., Laucou, V., Chatelet, P., Merdinoglu, D., Delledonne, M., Pezzotti, M., Lecharny, A., Scarpelli, C., Artiguenave, F., Pe, M.E., Valle, G., Morgante, M., Caboche, M., Adam-Blondon, A.F., Weissenbach, J., Quetier, F., Wincker, P. and French-Italian Public Consortium for Grapevine Genome, C. (2007) The grapevine genome sequence suggests ancestral hexaploidization in major angiosperm phyla. *Nature*, **449**, 463-467.
